# *DendroTweaks*: An interactive approach for unraveling dendritic dynamics

**DOI:** 10.1101/2024.09.06.611191

**Published:** 2024-09-10

**Authors:** Roman Makarov, Spyridon Chavlis, Panayiota Poirazi

**Affiliations:** 1 Institute of Molecular Biology and Biotechnology (IMBB), Foundation for Research and Technology-Hellas (FORTH), Heraklion, 70013, Greece; 2 Department of Biology, University of Crete, Heraklion, 70013, Greece

## Abstract

Neurons rely on the interplay between dendritic morphology and ion channels to transform synaptic inputs into a sequence of somatic spikes. Detailed biophysical models with active dendrites have been instrumental in exploring this interaction. However, such models can be challenging to understand and validate due to the large number of parameters involved. In this work, we introduce *DendroTweaks* — a toolbox designed to illuminate how morpho-electric properties map to dendritic events and how these dendritic events shape neuronal output. *DendroTweaks* features a web-based graphical interface, where users can explore single-cell neuronal models and adjust their morphological and biophysical parameters with real-time visual feedback. In particular, *DendroTweaks* is tailored to interactive fine-tuning of subcellular properties, such as kinetics and distributions of ion channels, as well as the dynamics and allocation of synaptic inputs. It offers an automated approach for standardization and refinement of voltage-gated ion channel models to make them more comprehensible and reusable. The toolbox allows users to run various experimental protocols and record data from multiple dendritic and somatic locations, thereby enhancing model validation. Finally, it aims to deepen our understanding of which dendritic properties are essential for neuronal input-output transformation. Using this knowledge, one can simplify models through a built-in morphology reduction algorithm and export them for further use in faster, more interpretable networks. With *DendroTweaks*, users can gain better control and understanding of their models, advancing research on dendritic input-output transformations and their role in network computations.

## 1 Introduction

Neurons are the most well-studied brain cells, known for their key role in processing and storing information. Information arrives from presynaptic axons via synaptic connections made onto neuronal dendrites, the extensive branching processes that receive signals from other neurons. Dendritic anatomy enables the segregation of incoming inputs. Additionally, the rich repertoire of ionic conductances that reside within dendrites enables the nonlinear integration of incoming signals, furnishing neurons with a wide range of input-output computations. Since Rall’s pioneering work on signal propagation within dendrites [Rall, 1959], the understanding of dendritic dynamics has expanded with the discovery of local regenerative events, such as Na^+^ dendritic spikes [Spencer and Kandel, 1961, Golding and Spruston, 1998], and NMDA and Ca^2+^ plateau potentials [Schiller and Schiller, 2001, Llinas and Nicholson, 1971, Llinás and Hess, 1976]. These active properties of dendrites depend on the interaction between their branching morphology and ion channel composition. Multicompartmental biophysical models with active dendrites have been instrumental in exploring this relationship. However, the problem with such models is that their numerous parameters complicate their interpretability, making them challenging to build, examine, and validate. In addition, the lack of standardization, along with their high computational complexity, makes these models less attractive for use in large-scale network simulations.

At the same time, with the advent of new techniques like genetic tracking of ion channels and high-resolution imaging of neuronal activity using voltage-sensitive dyes, multicompartmental biophysical modeling is experiencing its renaissance. Detailed biophysical models—although mathematically complex and computationally inefficient — are ideal for capturing and explaining data produced, for example, through simultaneous voltage and synaptic input imaging *in vivo*. Thus, to facilitate our understanding of how subcellular and dendritic processes impact neuronal output, new tools that can convert complex models into interactive visualizations that reflect changes in morpho-electric parameters in real time are needed. More specifically, it is necessary to provide interactive tools for working with kinetics and distributions of ion channels to unravel their interaction with dendritic morphology and their contribution to dendritic events. To our understanding, this is one of the biggest gaps in current neuronal modeling software. In addition, it is valuable to identify which dendritic properties are most important for neuronal function and which can be disregarded. Based on this understanding, one can simplify neuronal models and integrate them into faster, more interpretable networks. Thus, there is a need for a toolbox focused on subcellular and dendritic dynamics and covering multiple aspects of single-cell neuronal modeling, including fine-tuning of ion channel kinetics and distributions, morphology reduction, and validation of neuronal activity. Such a toolbox equipped with a modern, user-friendly graphical user interface (GUI) would make dendritic dynamics easier to comprehend and design on demand to reproduce specific single-neuron computations.

In this work, we introduce *DendroTweaks* — a toolbox designed to illuminate how morpho-electric properties of neurons map to dendritic events and how these dendritic events shape neuronal output. Building on existing methods, we have developed a comprehensive workflow for fine-tuning and validation of single-cell biophysical models with active dendrites. We aimed to achieve intuitive interaction with every parameter of the model and created a web-based GUI with a Python backend. The GUI allows interactive exploration, where any parameter of the model can be visually inspected and fine-tuned, with real-time feedback on the plots. Furthermore, we provide the means for statistical analysis and reduction of neuronal morphologies, standardization of ion channel models, and validation of somatic and dendritic dynamics. This approach results in more reusable, easier-to-understand models that are customizable according to the user’s needs. Overall, *DendroTweaks* makes input integration, signal propagation, and generation of local events in dendrites more intuitive and visually accessible and introduces an interactive approach for unraveling dendritic dynamics.

## 2 Results

### 2.1 Implementation and user interface

*DendroTweaks* is a Python-based toolbox designed for interactive exploration and fine-tuning of dendritic dynamics in detailed biophysical neuronal models. It is inspired by an exploratory data analysis approach and allows any user, from naive to expert, to gain an in-depth understanding of the model through interactive visualization. It utilizes the model-view-presenter (MVP) architecture, where a single-cell neuronal model implemented using the NEURON simulation environment [Hines and Carnevale, 2001] is presented through a web-based GUI built with the Bokeh library for data visualization [Bokeh Development Team, 2014]. The GUI is accessible via an online platform (https://DendroTweaks.dendrites.gr) or a locally hosted Bokeh server. Alternatively, a standalone Python library is available for direct interaction with the software’s core functionality. *DendroTweaks* accepts neuronal morphologies in .swc and .asc file formats, and ion channels models as .mod files, as input and outputs modified models in the same formats. Moreover, each session can be saved as a .json file and reuploaded when needed.

**Figure 1:**
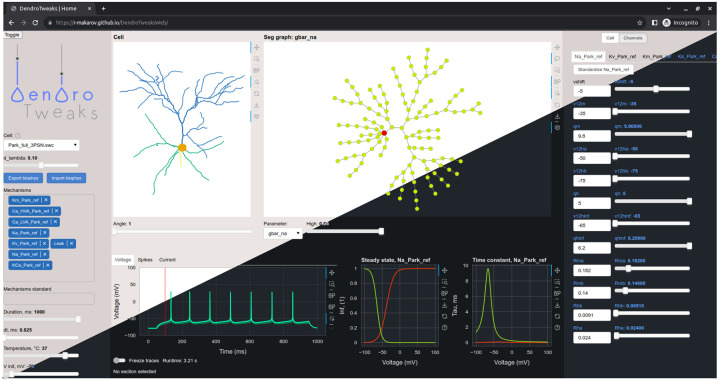
Graphical user interface (GUI). A screenshot of the web-based GUI accessed via the Chrome browser. The interface is organized into three main components: the left menu, the main workspace, and the right menu. It supports both light and dark themes.

The GUI is organized into three main components (Figure 1): (1) the left menu for file import and export operations, running simulation and validation protocols, and accessing application settings, (2) the main workspace with interactive plots, and (3) the right menu with widgets and conditionally toggled auxiliary plots. The main workspace contains top panels representing the cell and bottom panels representing monitors for the cell’s activity. In the top left corner, there is a morphology plot, where the uploaded morphology is rendered as a 2D projection of the cell. On the left of it, there is a graph representation of the cell’s computational segments, where different parameters, such as channel density or synapse allocation, can be visualized using a color code. The bottom panel can render time-dependent variables, such as voltage, current, and input spike times. In addition, this panel can display plots for channel kinetics. The right panel features widgets to manipulate cell morphology, distribution and kinetics of ion channels, as well as external current and synapses. Simulation parameters can be adjusted using widgets in the left menu. It is possible to activate real-time updates of the plots in the main workspace on interaction with widgets and the plots themselves.

The following sections will illustrate a potential workflow and provide a detailed description of the GUI elements and their functionality. We will start with exploring and refining dendritic morphology and choosing the spatial discretization of the model. Next, we will explore the kinetics of ion channels and propose an automatic algorithm for standardizing such models. We will also demonstrate how different distributions of these channels across the dendritic tree can be used to reproduce dendritic phenomena, such as sodium and calcium spikes. Next, we will demonstrate how synapses can be added to our models and explore how the kinetics and allocation of synapses can affect input integration within dendrites. At this point, we will be able to use an automatic morphology reduction algorithm in order to obtain a simpler model while retaining the activity close to the original one. Finally, we will present a set of experimental protocols that have been integrated in *DendroTweaks* to facilitate model validation.

### 2.2 Exploring dendritic morphology

**Figure 2:**
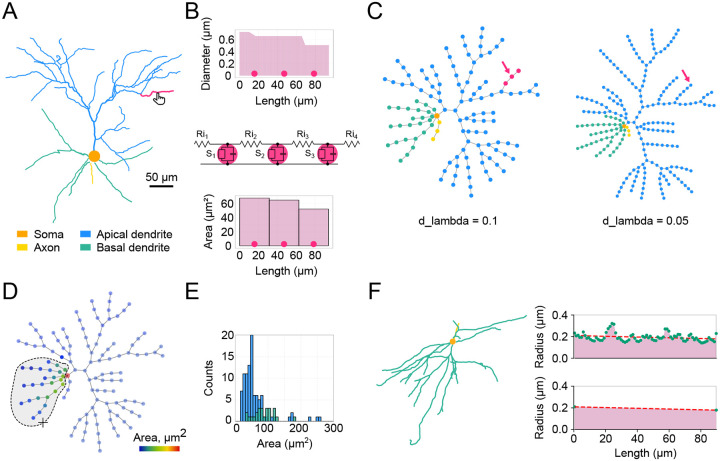
Dendritic morphology and segmentation. (**A**) Example of an L2/3 pyramidal neuron dendritic morphology from a .swc file. The selected section is highlighted in magenta; soma in orange, apical dendrites in blue, basal dendrites in green, and axon in yellow. (**B**) Detailed representation of the selected section. Top: Diameter of the selected section as a function of the section’s length, with circles marking segment centers. Middle: Equivalent circuit of the selected section shown as an RC circuit assuming a passive membrane. Bottom: Bar plot showing values of a user-selected parameter (surface area, $\mu m^{2}$) for each segment. (**C**) Segmentation network graph representing the same cell as in (A) with d_lambda parameters of 0.1 (left) and 0.05 (right); nodes represent segments, colored as in (A). (**D**) Visualization of the selected morphological parameter on the segmentation graph using a color code. The lasso mouse tool is shown, which allows the selection of specific segments. Statistical morphometric analysis can be performed for the selected part of the cell. (**E**) Histogram of segment areas for basal (green) and apical (blue) segments. (**F**) Example of dendritic geometry refinement for a somatostatin expressing (SST) interneuron. Left: Morphology of the SST neuron. Right: Original reconstructed diameter with plausible artifacts of reconstruction (top) vs. simplified diameter (bottom).

Developing a detailed biophysical neuronal model typically begins with the experimental reconstruction of a neuron’s morphology. Our toolbox accepts morphology reconstructions in the widely used .swc and .asc formats, which represent a cell as a collection of connected 3D points (XYZ coordinates and radius). A rich database of these files is readily available at https://neuromorpho.org [Ascoli et al., 2007]. Moreover, online conversion from all reconstruction formats to .swc and standardization of existing .swc files is possible [Mehta et al., 2023]. Upon uploading a neuronal morphology, it is rendered as a 2D (XY) projection of the cell in the main workspace on the top left Cell panel. This panel includes a slider for rotating the model around the Y-axis. For illustration, we use a realistic morphology of a Layer 2/3 (L2/3) pyramidal neuron of the mouse visual cortex [Park et al., 2019] (Figure 2 A). A neuron is typically divided into sections — parts of the cell between two bifurcation points. Users can navigate through the cell by selecting a section to visually inspect its parameters. This can be done by simply clicking on a section on the interactive plot or via a dropdown widget to select a specific section by its name. Navigation buttons are also available to select a parent, sibling, or child section. The parameters of the currently selected section are visualized to the left of the Cell panel, on a Section panel with two plots (Figure 2 B). The upper plot displays the geometry of the section, i.e., the diameter as a function of the section’s length. The lower plot shows a morphological or biophysical parameter selected by the user.

Computer simulations always involve approximating a continuous system as one that is discrete in space and time, which is also applied in neuronal modeling [Carnevale and Hines, 2006]. In NEURON, spatial discretization is achieved by dividing each section into segments. Each segment can be viewed as an equivalent RC circuit representing a part of the membrane, with an associated set of differential equations to calculate voltage dynamics at a given point in space and time (Figure 2 B, middle). In our example section, the centers of each segment are shown as three circles (nseg = 3), equally distributed along the section’s length according to the formula (2*i*–1)/2×nseg, where *i* is an integer in the range [1, nseg]. The bottom panel (Figure 2 B, bottom) features a bar plot showing the value for a chosen parameter (a NEURON range variable) for each segment. We also introduce a Graph view with a network graph representing all the segments of the given cell (Figure 2 C). This view serves three main purposes: (1) to visualize the distribution of different parameters along the dendritic tree using color code, (2) to select specific segments and update their parameters, and (3) to calculate statistics for a selected group of segments. By default, the graph plot uses color code to show different types of sections to which a segment belongs (same colors as in the Cell plot). Rendering other parameters on the graph is discussed in the following sections. The granularity of the graph depends on the number of segments. In addition to defining the number of segments (nseg) for each individual section, it is possible to select the d_lambda parameter in the left menu that automatically assigns the number of segments for each section based on the fraction of the length constant computed at the frequency of 100 Hz [Hines and Carnevale, 2001]. In the present example for the same neuron, we demonstrate a graph obtained with the default d_lambda value 0.1 (Figure 2 C, left) and with a finer spatial discretization using a value of 0.05 (Figure 2 C, right).

*DendroTweaks* supports basic morphometric analysis and morphology refinement. Statistical analysis applies to an arbitrary subset of segments selected by the user on the interactive plot (Figure 2 D). This analysis includes calculating the number of sections, segments, and bifurcations, along with average diameter, length, and area, total surface area, and total length. The number of root and leaf dendrites is displayed independently of the selection. An auxiliary histogram plot (Figure 2 E) further aids in visualizing the distribution of any calculated parameter. Additionally, the toolbox allows users to interactively inspect and refine morphological reconstructions. As an example, we present a case of an inhibitory interneuron from the Allen Institute database (https://celltypes.brain-map.org, ID 485466109) that exhibits variable diameters (Figure 2 F). These variations might represent real dendritic microgeometrical factors, such as dendritic varicosities [Korogod et al., 2023], or can be artifacts of morphological reconstruction. Users can identify these factors through visual exploration, and if they do not want to explicitly account for these details, they can “smooth” the diameters. Note that while microgeometry might not significantly affect the simulation at a low spatial resolution (small number of segments) due to averaging, it can introduce unexpected cell behavior at a higher level of spatial discretization.

### 2.3 Standardization and tuning of ion channel models

**Figure 3:**
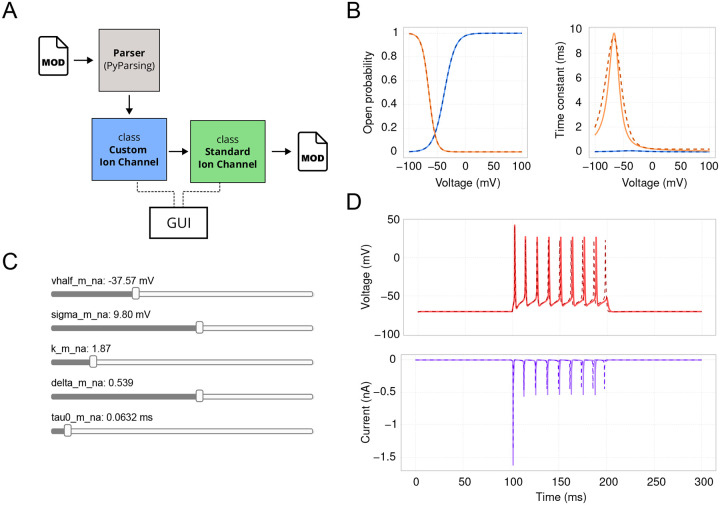
Standardization of ion channel models. (**A**) Schematic of parsing and standardizing ion channel models from mod files. A CustomIonChannel class is automatically generated and instantiated, facilitating interaction between the GUI and the channel model. This class includes a standardization method that applies an algorithm to produce a StandardIonChannel instance. The standardized channel model can then be saved to a new mod file. (**B**) Kinetics of a voltage-gated sodium channel. Activation (blue) and inactivation (orange) curves for the steady-state value (left) and the time constant (right). The dashed line represents the original model, while the solid line depicts the model with standardized equations fitted to the original curves. (**C**) Visualization of GUI sliders for adjusting the five standardized parameters of the activation gating variable *m*. (**D**) Corresponding voltage (top) and current (bottom) traces, with the current trace showing sodium current only. Note that the “missing” last spike can be restored by increasing the injected current amplitude by 0.01 pA.

In this section, we explore the biophysical properties of our model, starting at the individual ion channel level. Ion channels are crucial in shaping dendritic and somatic voltage dynamics, which are essential for neuronal communication and information processing. Since Hodgkin and Huxley’s seminal work [Hodgkin and Huxley, 1952], mathematical models have been vital in understanding channel kinetics [Petousakis et al., 2023a]. The most widely used ion channel models today are written in the NMODL domain-specific language developed for the NEURON simulator [Hines and Carnevale, 2000]. *DendroTweaks* features a custom parser written in PyParsing (https://pyparsing-docs.readthedocs.io), which parses .mod files and automatically creates a CustomIonChannel Python class representing a given ion channel (Figure 3 A). Upon importing a .mod file through the left menu and selecting the corresponding channel in the Channels tab on the right menu, two plots representing the channel’s kinetics appear in the workspace (Figure 3 B). The left plot shows the voltage-dependent steady-state asymptotic values of the channel’s gating variables, and the right plot shows the corresponding voltage-dependent time constants. When a channel is selected, interactive sliders for each parameter of the channel model appear in the right menu, allowing for the dynamic update of the plots.

Manual interaction with model parameters via the GUI is informative and provides good intuition about how each parameter affects voltage dynamics. However, it does not overcome the limitations of existing models. While valid and proven useful for exploring neuronal biophysics, many existing channel models exhibit inconsistencies and deviations from theoretical formulations, limiting their interpretability and reusability. Common issues include ambiguous variable names, inconsistent equations, hardcoded parameters, lack of units, incompatibility with the latest NEURON versions (such as problems with VERBATIM statements and *dt*), and potential overfitting to experimental data. These issues result in a steep learning curve for inexperienced modelers, a lack of proper exploratory analysis, laborious manual tuning of the models, and simulation errors.

Several efforts were made to ensure more efficient and consistent channel models. In 2010, Gleeson et al. [Gleeson et al., 2010] proposed standardizing models through NeuroML, an XML-based neuronal model description language, and manually converted models of voltage- and ligand-gated conductances using the ChannelML module. In 2017, Podlaski et al. [Podlaski et al., 2017] created a framework for the automated large-scale classification of ion channels, leading to the ICGenealogy web resource (https://icg.neurotheory.ox.ac.uk), which categorizes new and existing models and experimental recordings. In 2019, Kumbhar et al. [Kumbhar et al., 2020] introduced a framework that parses existing .mod files to generate optimized code, significantly improving simulation speeds. Recently, a comprehensive database of the voltage-gated potassium channel (Kv) family has been made available through Channelpedia (https://channelpedia.epfl.ch) and extended to include other channel types in the Channelome project [Ranjan et al., 2011, 2024]. However, none of these approaches offered an automatic, visual-guided standardization of existing .mod files through the standardization of model equations. Here, we utilize a standardization approach for .mod files using a set of equations grounded in transition state theory (see Materials and Methods).

To demonstrate the standardization procedure, we parsed and inserted voltage-gated sodium and potassium channels, and a leak channel into the cell’s membrane. We then ran a simulation for 300 ms with a step current injection (amplitude 500 pA, delay 100 ms, duration 100 ms, temperature 37^∘^C). Note that the parameters of the step-and-hold stimulation protocol (i.e., amplitude, delay, duration) can be adjusted using widgets in the Stimuli tab. For standardization, we selected the sodium channel, which has both activation (*m*) and inactivation (*h*) state variables (Figure 3 B). The standardization algorithm is initiated by clicking the “Standardize” button in the right menu and produces plots of the original (solid) and fitted (dashed) curves for steady-state values and time constants of the state variables (Figure 3 D). Each state variable in the standardized model has five parameters: v_half, sigma, k, delta, and tau_0, each with its own widget (Figure 3 C). Besides the activation curves, users can compare cell activity before and after standardization (Figure 3 D). To achieve this, there is an option to “freeze” the original current and voltage traces for comparison before running the standardization algorithm.

### 2.4 Distributing ion channels

Having explored and refined the neuronal morphology and individual ion channels, a user can proceed to set up the biophysical properties of their neuronal model, including the densities and distributions of the various ion channel conductances. Dendrites of most neuron types are known to express some types of active ion channels [Johnston and Narayanan, 2008], including major ion channel families such as Nav, Cav, Kv, KCa, and HCN [Yu et al., 2005]. The distribution of each channel type is cell type-specific and is often non-uniform. For example, CaV1.x channels, also known as high-voltage activated L-type channels, are densely populated in proximal dendrites [Reuveni et al., 1993, Westenbroek et al., 1990]. In contrast, CaV3.x channels, or low-voltage activated T-type channels, increase in density with distance from the soma [Magee and Johnston, 1995, Magee et al., 1995], forming “hot-spots” for dendritic Ca^2+^ spikes in the apical dendrite [Reuveni et al., 1993, Yuste et al., 1994]. Another compelling example is the hyperpolarization-activated mixed cation current (Ih), mediated by HCN channels, which are higher in density in distal apical dendrites of CA1 [Magee, 1998, Lörincz et al., 2002] and cortical Layer 5 (L5) [Kole et al., 2006] pyramidal neurons. Even passive conductances in dendrites can show non-uniform distribution [Stuart and Spruston, 1998]. While the distribution of dendritic ion channels has been the subject of significant research, there is still much to learn about their role in neuronal integrative function [Migliore and Shepherd, 2002, Johnston and Narayanan, 2008, Nusser, 2009, Shah et al., 2010]. Towards this goal, it is crucial to develop convenient tools for organizing ion channel distribution when designing software for studying neurons.

**Figure 4:**
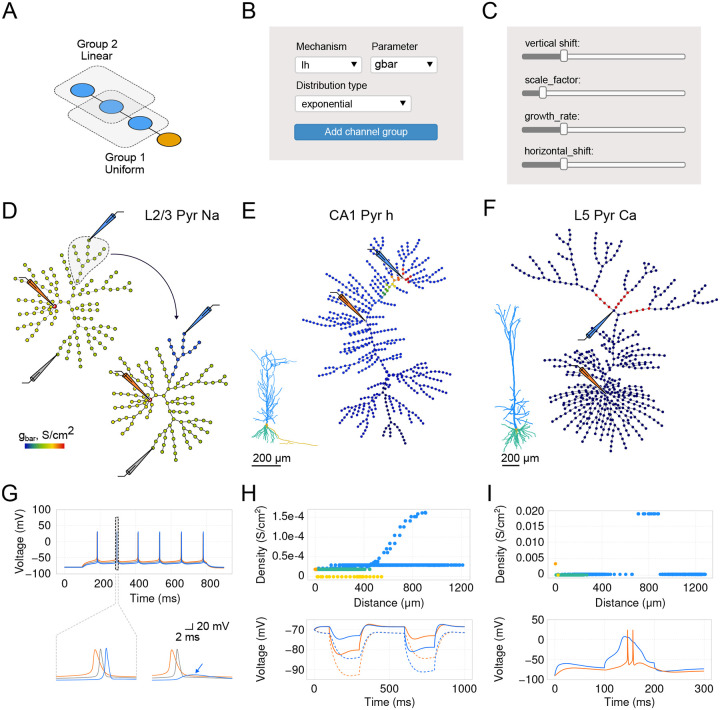
Distributions of ion channels. (**A**) Schematic of segment groups in a toy ball-and-stick model, each with a specific distribution as a function of distance from the soma. (**B**) Panel for creating a channel group. (**C**) Distribution parameters adjusted using the group’s widgets. (**D**) Graphs showing uniform Na channel conductance distribution (left) and a modified graph (right) where Na conductance in the selected region (dashed line) is decreased by 60%. Schematic electrodes indicate recording positions. Morphology from Park et al., 2019 [Park et al., 2019]. (**E**) Example of an exponential distribution for the HCN (Ih) channels (Inset - original morphology [Poirazi et al., 2003]). (**F**) Example of a calcium “hot spot” (red) (Inset - original morphology [Hay et al., 2011]). (**G**) Top: Sodium-driven backpropagation-activated action potentials (BAPs). A current step of 160 pA is applied at the soma. Bottom: Expanded time scale for the two scenarios in (D), showing failure of BAP spike initiation (blue arrow) in the region with decreased sodium conductance. (**H**) Top: Distribution of maximal conductance of HCN channels as a function of distance from the soma. Bottom: Voltage sag produced by HCN channels. Current injected at the somatic (−200 pA, 200 ms) and then, after 300 ms, at the dendritic electrode at the distal apical trunk (697 *μm* from the soma). Dashed trace: blocking HCN channels, modeled as 80% decrease in channel conductance. (**I**) Top: Distribution of maximal conductance of calcium channels as a function of distance from the soma. Bottom: A dendritic calcium plateau potential triggered by dendritic step current injection (500 pA, 100 ms) at the calcium “hot-spot”, leading to somatic firing. Somatic traces are shown in orange, dendritic in blue (and gray).

To make the process of exploring and adjusting the distributions of membrane mechanisms more user-friendly and intuitive, we utilize the graph view introduced earlier. Every membrane mechanism (distributed mechanism in NEURON) can be visualized on the graph, such that its value for a given segment is color-coded. In *DendroTweaks*, parameters for each membrane mechanism are specified for groups of segments (Figure 4 A). Each group is associated with a distribution defined as a function of distance from the soma. To create a new MechanismGroup, users must select a mechanism (e.g., a sodium channel), its parameter (e.g., maximal conductance, gbar), and a type of distribution (Figure 4 B). There are several built-in types of distributions (i.e., uniform, linear, exponential, sigmoidal, and normal) which, when combined, can cover most of the existing models. Parameters of a given distribution are controlled by distribution widgets (Figure 4 C).

To illustrate the effectiveness of this interface in distributing dendritic mechanisms and investigating their impact on neuronal activity, we replicated several established models. First, we reproduced the L2/3 pyramidal neuronal model from Park et al., 2019 [Park et al., 2019], to show dendritic sodium-driven backpropagation-activated action potentials (BAPs, Figure 4 D, G). Using this model, we were able to demonstrate that the distribution of sodium channels affects dendritic BAPs, as removing sodium channels from a specific branch prevented spike generation in that branch. Next, we replicated the CA1 pyramidal neuronal model from Poirazi et al., 2003 [Poirazi et al., 2003] to demonstrate the effect of the experimentally observed exponential distribution of HCN channels (Figure 4 E, H). As observed in the original study, we noted a significant depolarizing voltage sag, which was eliminated by blocking HCN channels. Finally, we replicated the model of an L5 pyramidal neuron from Hay et al., 2011 [Hay et al., 2011] to demonstrate dendritic Ca^2+^ spikes. We were able to evoke a dendritic Ca^2+^ plateau potential by injecting current at the Ca^2+^ “hot-spot” of the apical dendrite (Figure 4 F, I). This plateau potential then propagated to the soma, resulting in somatic burst firing. Overall, by replicating these models and respective simulations, we demonstrate how *DendroTweaks* can aid in investigating the role of ion channels in generating dendritic events through interactive parameter adjustment and visualization.

### 2.5 Distributing synapses

All previous examples used simple stimuli such as somatic and dendritic step current injections to demonstrate the functionalities of *DendroTeaks*. In this section, we describe how more realistic synaptic stimulation protocols can be implemented. Synaptic allocation and timing play an important role in the integration of synaptic inputs. For example, neocortical pyramidal neurons respond supralinearly to spatially clustered inputs and sublinearly to randomly distributed ones [Polsky et al., 2004, Losonczy and Magee, 2006, Takahashi et al., 2012, Poirazi et al., 2003, Tran-Van-Minh et al., 2015]. Another layer of complexity to synaptic integration is added by the interplay between excitatory and inhibitory inputs [Doron et al., 2017, Du et al., 2017]. Interestingly, connections from different types of inhibitory interneurons target different dendritic domains of pyramidal neurons [Markram et al., 2004, Van Versendaal and Levelt, 2016], allowing for the selective regulation of information streams within a neuron. In light of the above, the ability to reproduce various synaptic input patterns is crucial for understanding dendritic integration.

**Figure 5:**
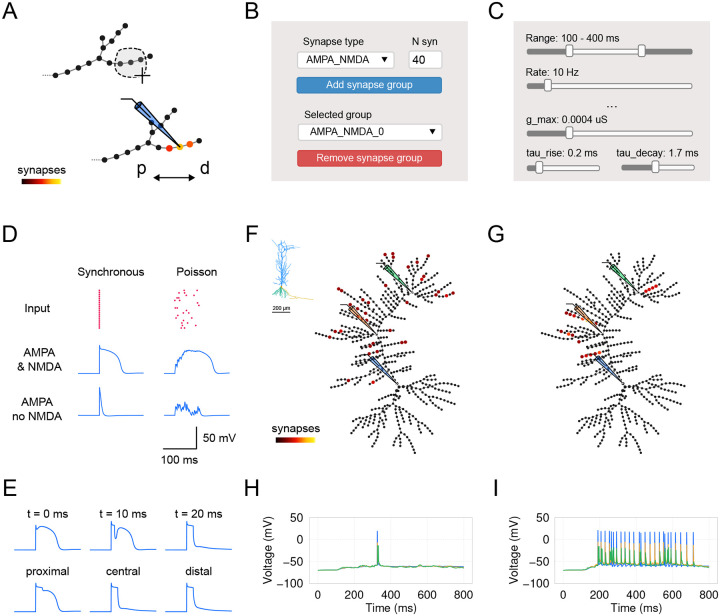
Kinetics and distribution of synapses. (**A**) Schematic representation of distributing synaptic inputs. Three central segments of a distal apical branch are selected using the lasso tool, and synapses are added. p — proximal, d — distal. (**B**) Panel for associating a synapse group with the selected segments. The user must specify the type and number of synapses in a group. (**C**) The kinetics of synapses and input properties can be further adjusted using the group’s widgets. Note that not all available widgets are shown for visualization purposes. (**D**) Example responses evoked by activating 20 excitatory synapses placed within one branch as in (A). The regularity of inputs varies from synchronous activation to a random Poisson spike train. Note that the raster plot for input times is accessible in one of the workspace tabs. The examples demonstrate dendritic voltage responses in the presence or absence of NMDA conductances. (**E**) Experiment similar to Doron et al., 2017 [Doron et al., 2017], demonstrating the effect of inhibiting NMDA spikes. Top: One inhibitory GABA_*A*_ synapse is placed in the middle of the section and its activation time varies as 0, 10, and 20 ms after excitatory synapse activation. Bottom: The synapse location varies from the most proximal to the most distal segment of the section, with the activation time kept at 20 ms. The same stimulation protocol as in (D) with synchronous activation is used for the excitatory inputs. Scale is the same as in (D). Distributed (**F**) and clustered (**G**) allocation of 40 excitatory AMPA-NMDA synapses, similar to an experiment from Poirazi et al., 2003 (Inset - original morphology, [Poirazi et al., 2003]). (H) Distributed synaptic inputs ( 10 Hz Poisson) nearly fail to evoke somatic action potentials. (I) High firing activity evoked by the same exact synapses clustered within five randomly selected branches.

As we do for ion channels, we use the graph view to visualize and allocate groups of synaptic inputs. To create a SynapseGroup, users must select segments (Figure 5 A) and define group parameters using the dedicated widgets in the right menu (Figure 5 B). The creation of a group requires users to select a synapse type and specify the number of synapses to be uniformly distributed randomly within the selected segments. *DendroTweaks* offers three built-in synapse types: AMPA, NMDA, and GABA_*A*_, with an additional option for a combined AMPA-NMDA synapse. Users can create as many groups as necessary. Each group can have unique kinetic parameters for the synapse (maximal conductance, g_max, equilibrium potential, e, time constants tau_rise, tau_decay) as well as parameters for incoming inputs (input rate, randomness/noise, onset, duration). Parameter values can be adjusted using the group sliders (Figure 5 C).

To demonstrate the power of *DendroTweaks* for exploring dendritic integration of synaptic inputs, we conducted *in silico* experiments involving different allocations and activation times of synaptic inputs. First, we examined the effect of input synchronicity and NMDA synapses on generating NMDA spikes (Figure 5 D). Using the graph view, we distributed 20 excitatory AMPA-NMDA synapses within a single section of the Hay et al., 2011 passive model [Hay et al., 2011]. We observed that simultaneous activation of synapses or activation with a Poisson spike train produced distinct dendritic voltage responses. Additionally, by blocking NMDA conductances, we were able to eliminate NMDA spikes. Second, we replicated the effect of inhibition on dendritic NMDA spike generation, as shown in Doron et al., 2017 [Doron et al., 2017]. We added one inhibitory GABA_*A*_ synapse in the same section and varied its activation time or location (Figure 5 E). Consistent with the original study, NMDA spikes could not be recovered if inhibition occurred 20 ms after excitation. Moreover, proximal inhibition had little effect on NMDA spikes, whereas distal inhibition significantly reduced them. Finally, we conducted an experiment similar to that described in Poirazi et al., 2003 [Poirazi et al., 2003], using the CA1 pyramidal neuronal model with active dendritic mechanisms. Using the graph view, we distributed 40 excitatory AMPA-NMDA synapses in two scenarios: either spread across multiple terminal branches of the apical dendrite (Figure 5 F) or clustered within five randomly selected branches of the apical dendrite (Figure 5 G). As in the original study, we found that distributed synapses failed to evoke high-frequency somatic activity (Figure 5 H), whereas clustering synapses made high-frequency somatic activity possible (Figure 5 I). These experiments illustrate how *DendroTweaks* can facilitate the investigation of synaptic input integration and its effects at the dendritic and somatic levels.

### 2.6 Reducing morphology

The explorations described in the previous sections aimed to enhance the user’s understanding of which dendritic properties are essential for specific neuronal input-output transformations. With this knowledge, one can then proceed to simplify models using a built-in morphology reduction algorithm and export them for further use in faster and more interpretable neuronal networks.

Here, we follow the analytical impedance-based approach proposed by Amsalem et al., 2020 (neuron_reduce) [Amsalem et al., 2020]. This method maps a detailed dendritic tree to an equivalent cylinder with the same passive properties (i.e., specific membrane resistivity, capacitance, and axial resistivity). The transfer impedance from the distal sealed end to the soma in the simplified model matches the transfer impedance from the most distal dendritic tip to the soma in the detailed model. Additionally, the input impedance at the proximal end matches that of the respective detailed dendrite when decoupled from the soma. In the original implementation, the entire subtree of each stem dendrite (e.g., the entire apical subtree) is mapped to a single corresponding cylinder. This, however, can impose some limitations on accurately capturing the complex branching patterns and electrotonic properties of the dendritic tree, potentially affecting the precision of simulations of synaptic integration and signal propagation.

**Figure 6:**
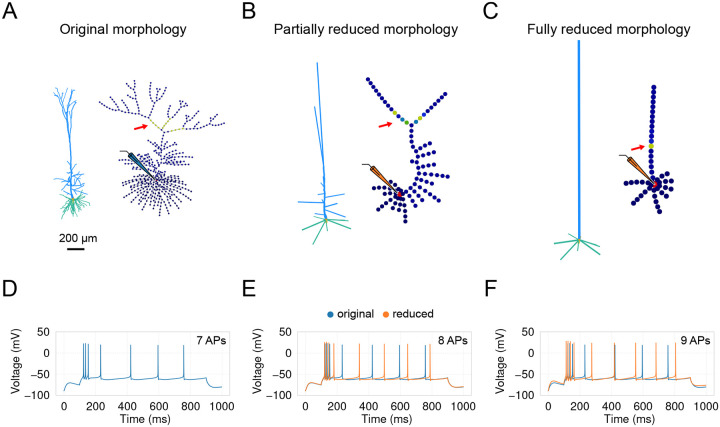
Morphology reduction. (**A**) Original morphology of L5 pyramidal neuron [Hay et al., 2011] and its segmentation graph. (**B**) Partially reduced morphology using the extended version of neuron_reduce. The extended version allows for the reduction of any selected branch, allowing to retain more apical branches, in contrast to (C). (**C**) Fully reduced morphology. All stem dendrites (children of the soma) are reduced to a single equivalent cylinder. (**D–F**) Voltage response of the three models to somatic current injection of 500 pA. Note the difference in the number of somatic APs between the three variations of the model. The partially reduced model (E) more accurately reproduces the response of the original model (D) compared to the fully reduced one (F).

In *DendroTweaks*, we extended the functionality of neuron_reduce to allow for a continuum of morphology reduction levels, bridging detailed and “ball-and-stick”-like models. Users can select any section of the cell and reduce its subtree, which allows for any intermediate level of detail to be achieved. As an example, we start with the Hay et al., 2011 [Hay et al., 2011] model used in the original study (Figure 6 A). We reduce its morphology using the original algorithm to the “ball-and-stick” level (Figure 6 C) and to an intermediate level where some apical oblique and tuft dendrites are preserved (Figure 6 B). Notably, the response of the partially reduced model (Figure 6 E) is closer to the original model’s response (Figure 6 D) in terms of the number of spikes compared to the response of the fully reduced model (Figure 6 F).

By integrating an enhanced version of neuron_reduce into *DendroTweaks*, we ensure easier post-reduction fine-tuning of model parameters. The graph view allows users to visualize the resulting distributions of channels and synapses after they have been mapped onto the reduced morphology. The simplified model can be re-validated to ensure it faithfully reproduces experimental observations. In the next section, we will discuss several built-in validation protocols that can be used for both original and simplified models.

### 2.7 Validating biophysical properties

Thus far, we have presented a comprehensive set of tools available in *DendroTweaks* for developing and exploring the parameters of multicompartmental single-cell biophysical models. In addition to these functionalities, *DendroTweaks* also offers some built-in validation protocols that allow users to ensure the resulting models align with experimental observations. This approach is semi-automated, requiring users to manually implement a stimulation protocol by setting the stimulation parameters. For example, to validate somatic action potentials, a user must apply a positive step current injection at the soma to produce somatic firing. In the Validation panel of the left menu, selecting the Detect APs command will automatically detect action potentials and measure their properties, such as firing rate, amplitude, and half-width. While this process is not fully automated, it allows for the use of custom stimulation protocols instead of relying on predefined stimuli parameters, thus offering more flexibility.

**Figure 7:**
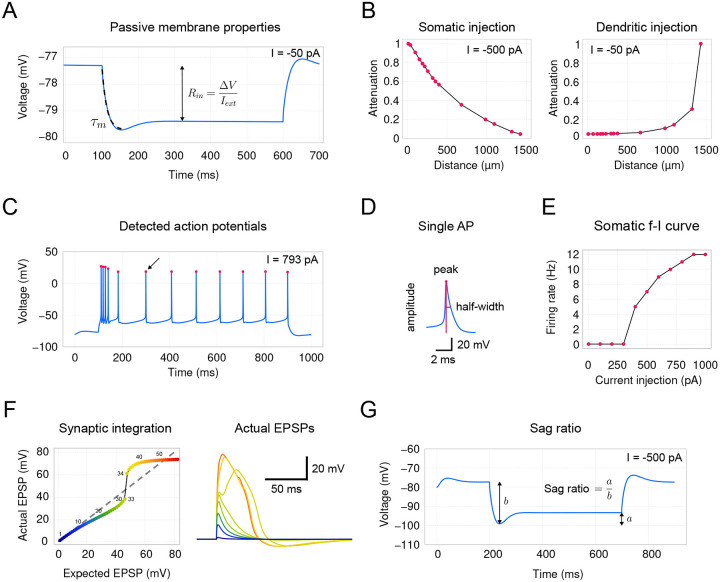
Validation protocols. Build-in validation protocols applied to the Hay et al., 2011 model [Hay et al., 2011]; See also Table 1). (**A**) Validation of passive membrane properties. Input resistance (42 MOhm) and membrane time constant (13 ms) measured by applying a step current injection (−50 pA) at the soma. (**B**) Voltage attenuation for somatic ( −500 pA, left) and dendritic (−50 pA, right) step current injection at all bifurcation points along the path from a selected tip segment. (**C**) Detected somatic action potentials from stimulation with a positive step current (793 pA). (**D**) Single action potential indicated with an arrow in (C), with measured peak, amplitude, and half-width values. (**E**) Somatic frequency-current (f-I) curve constructed by applying current steps of increasing amplitude (100 pA step) at the soma. (**F**) Nonlinear integration of synaptic inputs in a tuft dendrite. Left: Expected vs. actual EPSP amplitude for 1 to 60 synchronously activated AMPA-NMDA synapses. Right: Actual EPSP waveforms. (**G**) Voltage sag ratio at the soma measured by applying a negative step current injection (−500 pA).

We demonstrate validation of passive and active properties using built-in protocols applied to the Hay et al., 2011 model [Hay et al., 2011]. First, we validate input resistance (42 MOhm) and membrane time constant (13 ms) (Figure 7 A) by applying a step current injection (−50 pA) at the soma. We then measure voltage attenuation for either somatic (−500 pA, Figure 7 B , left) or dendritic (−50 pA, Figure 7 B, right) step current injection. Next, we stimulate the soma with a positive step current (793 pA) and detect somatic action potentials (Figure 7 C). From the same trace, we measure the peaks, amplitudes, and half-widths of individual action potentials (Figure 7 D). The somatic output statistics (mean rate, interspike interval (ISI), etc.) are shown to the user in the Validation panel. We then construct a somatic f–I curve (Figure 7 E) by applying current steps of increasing amplitude (100 pA step). After validating the somatic activity, we evaluate dendritic integration nonlinearity (Figure 7 F, left) by comparing measured individual waveforms (Figure 7 F, right) to the expected linear summation of postsynaptic potentials (PSPs). Note that protocols for both the somatic f–I curve and dendritic integration curve require multiple simulation runs with varying stimulus intensity, which are done automatically after the user specifies the initial stimulation parameters. Finally, since the model includes Ih current, we measure the voltage sag ratio by applying a negative step current injection (−500 pA) at the soma (Figure 7 G). For a detailed description of the protocols, see Table 1 in Materials and Methods.

## 3 Discussion

### 3.1 An interactive approach for unraveling dendritic dynamics

The main motivation for developing *DendroTweaks* was to illuminate how morpho-electric properties map to dendritic events and how these dendritic events shape somatic firing. Such an understanding remains elusive due to the complexity of dendritic geometry and channel distributions, which challenge our intuitive grasp of dendritic dynamics. While significant progress has been made in understanding dendritic dynamics through the discovery of local dendritic spikes and plateau-like events [Mel, 1994, Häusser and Mel, 2003, Poirazi and Papoutsi, 2020, Larkum et al., 2022], a comprehensive “periodic table” that systematizes dendritic properties by mapping them onto specific computations has yet to emerge. As a result of our limited understanding, many state-of-the-art network models still consider dendrites as passive cables [Markram et al., 2015, Billeh et al., 2020], greatly underestimating their computational power [Tran-Van-Minh et al., 2015]. To unravel the full beauty of dendritic dynamics and make this knowledge accessible to a wider audience, it is essential to develop community-driven tools specifically designed to tackle these challenges.

Towards this goal, we have developed a comprehensive toolbox for single-cell modeling, accessible through an interactive web-based graphical interface. Inspired by the exploratory data science approach, we equip a neuronal model with widgets and interactive plots, making every parameter of the model visually accessible and interactively tunable. Importantly, for simple models, plots respond to user actions in real time, ensuring a smooth model exploration and tuning process. Interactive plots illustrating neuronal morphology significantly simplify the process of navigating through various sections and segments of a model. In addition to visual exploration, we provide the means for refinement, standardization, and reduction of model parameters. We focus on making ion channel models more interactive by providing an interface and automatic standardization to .mod files. Furthermore, by representing a cell as a graph with computational segments as nodes, we simplify and visually enhance the process of distributing ion channels throughout the cell. Finally, we extended the neuron_reduce approach for morphology reduction to encompass all potential reduction levels from full morphology to “ball-and-stick”-like models and incorporate this method into our graphical interface, thereby taking advantage of inherent visualization and validation capabilities.

### 3.2 Comparison to existing modeling software

Single-cell modeling encompasses a diverse range of practices, from refining morphological data and optimizing biophysical properties to visualizing and analyzing neuronal dynamics. Over the years, numerous tools have been developed to aid in the creation, visualization, and optimization of neuronal models. Primary simulation environments like NEURON [Hines and Carnevale, 2001] and BRIAN2 [Stimberg et al., 2019] have been complemented by a variety of auxiliary software tools designed to enhance interaction with model parameters.

Several tools have been developed to assist in visualizing and editing morphological reconstructions of real neurons, such as neuTube [Feng et al., 2015], REMOD [Bozelos et al., 2016], HBP MORPHOLOGY VIEWER [Bakker et al., 2017], and HUGO [Aliaga Maraver et al., 2018]. *DendroTweaks* offers the possibility for inspecting and refining morphological parameters to ensure the development of robust models and identify artifacts and bugs through visual exploration and statistical analysis. Nevertheless, it lacks advanced tools like 3D mesh editing and neuronal growth modeling capabilities. For more extensive morphology-focused needs, users are directed to specialized software like the TREES toolbox [Cuntz et al., 2011], Neuronize [Brito et al., 2013], NeuroEditor[Velasco et al., 2024], and NETMORTH [Koene et al., 2009].

Another critical aspect of neuronal modeling is parameter optimization. Evolutionary [Van Geit et al., 2016], Bayesian [Gonçalves et al., 2020], and gradient-based methods [Jones and Kording, 2024] have been proposed to offer data-driven model parameter optimization. While saving time and effort, automatic optimization can obscure the impact of individual parameter changes on the model output. *DendroTweaks* does not aim to replace automatic optimization with manual adjustments but emphasizes the importance of an exploratory approach for model development and debugging. Incorporating automatic optimization algorithms into *DendroTweaks* alongside interactive visualizations presents a promising future direction, combining the strengths of both approaches.

With a growing number of models available online from repositories like https://modeldb.science [McDougal et al., 2017], https://neuromorpho.org [Ascoli et al., 2007], and https://celltypes.brain-map.org, standardization is crucial for ensuring reproducibility and reusability in neuronal modeling. Existing standards like SONATA [Dai et al., 2020], NeuroML [Gleeson et al., 2010], and NineML [the INCF Multiscale Modeling Taskforce and Gorchetchnikov, 2010] provide frameworks for model description. However, manual standardization can be laborious and error-prone. Importantly, *DendroTweaks* is not presented as a new standard but as a tool that conforms to a given standard to automatically standardize a model. Future releases might include an automatic export to an expanded range of formats, such as ChannelML [Gleeson et al., 2010]. Finally, to our knowledge, *DendroTweaks* is the only tool that can simultaneously parse existing ion channel model files, allow for visual exploration and fine-tuning of their kinetics and distributions via a GUI, and offer automatic standardization.

A universal workflow for creating, validating, and generalizing detailed neuronal models has been proposed by Reva et al., 2023 [Reva et al., 2023]. This workflow uses electrophysiological features during the optimization phase with BluePyOpt, followed by validation to ensure model accuracy. BluePyMM then assesses the morphological generalizability of electrical models across diverse morphologies. While robust and beneficial for large-scale simulations, this approach does not focus on in-depth exploration and fine-tuning at the subcellular level. *DendroTweaks*, with its emphasis on channel kinetics and dendritic dynamics, complements these methods to address different aspects of neuronal modeling.

Visualization and the development of intuitive graphical user interfaces have been crucial in neuronal modeling. One of the most successful examples is NetPyNE [Dura-Bernal et al., 2019], which offers a graphical interface for data-driven multiscale network modeling in NEURON. In contrast, *DendroTweaks* focuses on the subcellular level, providing a more explicit single-cell model interface. It facilitates an interactive approach to visualize and modify morphological parameters, ion channel kinetics, and distributions, as well as to observe activity in different compartments. This capability is particularly important for models with active dendrites. We envision as a good practice exporting fine-tuned single-cell models from *DendroTweaks* and incorporating them into complex networks in NetPyNE.

Indeed, the ultimate goal of creating single-cell models is often to incorporate them into a network. In this context, simplifying models becomes another crucial aspect of neuronal modeling. Morphology reduction is a common simplification technique with a long history. In pioneering works, Stratford et al., 1989 and Bush and Sejnowski, 1992 [Stratford et al., 1989, Bush and Sejnowski, 1993] proposed a method based on conserving axial resistance, reducing multicompartmental models to 8–9 compartments. Later, different approaches focused on preserving voltage attenuation [Destexhe, 2001] or surface area [Hendrickson et al., 2011, Marasco et al., 2013]. The most recent methods provide analytical solutions to the reduction problem by preserving the impedances of the original model. For example, Wybo et al., 2020 [Wybo et al., 2021] suggested a technique where conductance matrices of the reduced model fit the impedance matrices of the original model for a set of holding potentials, and capacitances of the reduced model match the temporal dynamics of the full model.

For neurons, having a dendritic tree offers the advantage of semi-independent input integration across distinct subunits, enabling localized signal processing and enhancing computational complexity. Spatial organization of inputs plays a crucial role in their integration, with clustered inputs being more likely to drive somatic firing in pyramidal neurons [Poirazi et al., 2003, Polsky et al., 2004, Losonczy and Magee, 2006]. When multiple synaptic inputs from otherwise isolated branches are remapped to a reduced cylinder, clustering can occur, substantially altering the integration process. Therefore, the intermediate levels of reduction we added to neuron_reduce could potentially allow for more accurate implementation of independent input integration within dendritic subunits. A compelling example of when this might be needed is recent research by Otor et al., 2022 [Otor et al., 2022], which showed that early bifurcating L5 pyramidal neurons exhibit pronounced functional compartmentalization of the apical dendrite, correlating with behavioral variables. Nevertheless, it is still not clear whether the computational unit of a neuron is a single spine, a branch, or entire basal and apical domains [Häusser and Mel, 2003, Francioni and Harnett, 2022, Stuyt et al., 2022]. We highlight that, aside from optimizing simulation speed, morphology reduction is a useful technique to explore the importance of dendritic compartmentalization. Using morphology reduction at multiple levels in *in silico* experiments can help address different cell types and computations, shedding light on how morphology affects compartmentalization and the neuron’s input-output transformation.

### 3.3 Limitations and further directions

A key limitation of the current *DendroTweaks* implementation is the range of ion channel models that can be standardized. As of now, only voltage-gated channels using the Hodgkin-Huxley (HH) formalism can be standardized. Even though Markov chain state-based kinetic models might offer a more accurate representation of ion channel kinetics [Lampert and Korngreen, 2014], they are not supported by *DendroTweaks*. Nevertheless, it is important to note that most models use the HH formalism as Markov models can be more complex and slower to run. Additionally, our parsing and automated standardization algorithm relies on specific heuristics and cannot handle significant deviations in .mod file code patterns. For example, the parser assumes that variable names for the time constant will include “tau” (case insensitive). Therefore, for some files the algorithm may require some minor preprocessing and manual tuning by the user.

*DendroTweaks* is designed to read and write the most popular formats for representing neuronal morphology, namely .swc and .asc, as well as ion channel models, i.e., .mod files. To enhance compatibility with existing models and the reusability of standardized models, support for more file formats and standards needs to be incorporated. One notable example is NeuroML [Gleeson et al., 2010], an XML-based neuronal model description language that provides a standardized notation for both morphological and biophysical parameters. By extending the range of file types that *DendroTweaks* can handle, it can be more seamlessly integrated into a larger ecosystem of interoperable, open-source software [Sinha et al., 2024].

The performance of *DendroTweaks* in its current implementation is constrained by the limitations of the NEURON simulator. When conducting hyper-detailed simulations with numerous segments over extended periods of time, the simulation can become slow, reducing the interface’s responsiveness and real-time update capabilities. To enhance performance, future versions of *DendroTweaks* should consider integrating faster simulation methods or developing a custom single-cell simulator. A promising approach is to run simulations using the optimized CoreNEURON [Kumbhar et al., 2019] and Dendritic Hierarchical Scheduling (DHS) algorithms [Zhang et al., 2023], which have been shown to greatly speed up NEURON simulations. Alternatively, support for the NeuroML-based neural simulator EDEN can be added, which was shown to be two orders of magnitude faster than NEURON [Panagiotou et al., 2022]. This increase in speed would allow for real-time, interactive simulations of high-resolution cell models with multiple membrane mechanisms and realistic stimuli.

Importantly, while the current implementation of *DendroTweaks* is based on the NEURON simulator, the approach is essentially simulator-agnostic. Its functionality can be further extended to include other simulation engines. One of the promising future directions is to include integrate-and-fire few-compartmental models implemented in BRIAN2 using Dendrify [Pagkalos et al., 2023]. This would allow for the extension of the proposed workflow by automatically simplifying not only the morphology but also the biophysics of a neuron, spanning any level of conceptual granularity.

### 3.4 Conclusion

We believe that *DendroTweaks* will be appealing to a wide range of researchers. For those who are new to computational modeling, it offers an intuitive understanding of how model parameters affect neuronal dynamics, making it also a valuable educational tool. Its web-based interface eliminates the need for local simulations, making modeling more accessible. With the increasing focus on dendritic computations in artificial intelligence and neuromorphic computing, *DendroTweaks* offers clear visualizations of dendritic integration, benefiting those less familiar with biophysical modeling. For experienced modelers, *DendroTweaks* provides a complete workflow, from single-channel analysis to validating dendritic properties, with tools for visual inspection of cell topology, geometry, channel kinetics, and their distributions, as well as neuronal activity under variable stimuli, aiding in visual debugging. It also includes standardization and morphology reduction tools to improve model tractability and reusability for network simulations. *DendroTweaks* will evolve with community feedback, adding new features over time. More than just a tool, *DendroTweaks* is a versatile framework that can integrate other visualizations and algorithms, ensuring its lasting relevance in the research community.

## 4 Materials and Methods

### 4.1 User interface

The user interface of *DendroTweaks* is implemented using Python 3, following the Model-View-Presenter (MVP) architectural pattern. The core of the application is the Model, a Python class that contains a biophysical model created with NEURON [Hines, 2009]. For the View class, we use the Python Bokeh library [Bokeh Development Team, 2014], which facilitates data visualization by generating the necessary JavaScript code to build the web-based interface. The Presenter is a class that acts as an intermediary, processing user commands, updating the Model accordingly, and ensuring that the View reflects the current state of the Model. This separation of concerns ensures a clean and maintainable codebase, allowing for efficient data handling and user interaction.

### 4.2 Biophysical models

To demonstrate the capabilities of the toolbox, we employed three well-established biophysical neuronal models with detailed morphology. The first model is an L2/3 pyramidal neuronal model with morphology reconstruction from Park et al., 2019 and biophysical mechanisms originally developed by Mainen et al. [Mainen and Sejnowski, 1996], further refined by Smith et al. [Smith et al., 2013], and recently utilized in Park et al. [Park et al., 2019] and Petousakis et al. [Petousakis et al., 2023b]. The membrane potential was initialized at $V_{\text{init}}=-79\;\text{mV}$, and simulations were conducted at 37^∘^C. The equilibrium potentials were set as follows: $E_{\text{leak}}=-79\;\text{mV},\;E_{\text{na}}=60\;\text{mV},\;E_{\text{k}}=-80\;\text{mV}$, and $E_{\text{ca}}=140\;\text{mV}$. The second model is a widely-used L5 pyramidal neuronal model with morphology reconstruction from Amitai et al. [Amitai et al., 1993] and biophysical mechanisms from Hay et al. [Hay et al., 2011]. For this model, the membrane potential was initialized at either $v_{\text{init}}=-80\;\text{mV}$ or $V_{\text{init}}=-90\;\text{mV}$, with simulations performed at 37^∘^C. The equilibrium potentials were $E_{\text{leak}}=-90\;\text{mV},\;E_{\text{na}}=50\;\text{mV},\;E_{\text{k}}=-85\;\text{mV}$, and $E_{\text{ca}}=132\;\text{mV}$. The third model is a CA1 hippocampal pyramidal neuron, based on Poirazi et al. [Poirazi et al., 2003] and [González, 2011]. Here, the membrane potential was initialized at $V_{\text{init}}=-70\;\text{mV}$, with simulations conducted at 34^∘^C. The equilibrium potentials were $E_{\text{leak}}=-70\;\text{mV},E_{\text{na}}=50\;\text{mV}$, $E_{\text{k}}=-77\;\text{mV}$, and $E_{\text{ca}}=140\;\text{mV}$. All simulations were executed on a Dell G15 5515 laptop (Ryzen 7 5800H, 16GB RAM, Linux Ubuntu 20.04 LTS) with a spatial discretization factor $d_{-}\lambda =0.1$ and a time step *dt* = 0.025 ms.

### 4.3 Standardization of ion channel models

Voltage dynamics in a given segment are determined by the RC circuit differential equations. These equations describe the time derivative of the law of capacitance Q=CV (first Kirchhoff’s law) and consider both capacitive (left) and resistive (right) currents.

(1) $$C\frac{dV}{dt}=\sum_{i=1}^{n}I_{i}\left(t\right)$$

where:

V — the membrane potential in mV

C — the membrane capacitance in μF

Ii — current in pA

t — time in ms

For a given ion channel, the current I is described by:

(2) $$I=g\times p\left(x_{1},\cdots ,x_{n}\right)\times \left(V-E\right)$$

where:

g — the channel conductance in S

$x_{i}$ — a state variable

p — the function defining the probability of the channel to be open (e.g. for an HH sodium channel $p\left(m,h\right)=m^{3}h$

E — the equilibrium potential in mV

The time derivative of a state variable x is given by:

(3) $$\frac{dx}{dt}=\frac{x^{\infty }-x}{\tau_{x}}$$

where:

$x^{\infty }$ — the steady-state value of x (unitless)

$\tau_{x}$ — the time constant in ms

The steady-state value $x^{\infty }$ is defined as:

(4) $$x^{\infty }=\frac{1}{1+\text{exp}\left(-\frac{V-V_{\text{half}}}{\sigma }\right)}$$

The time constant $\tau_{x}$ is given by:

(5) $$\tau_{x}=\frac{1}{\frac{d\alpha }{dt}+\frac{d\beta }{dt}}+\tau_{0}$$

where:

(6) $$\frac{d\alpha }{dt}=K\times \exp\left(\frac{\delta \times \left(V-V_{\text{half}}\right)}{\sigma }\right)$$

(7) $$\frac{d\beta }{dt}=K\times \text{exp}\left(\frac{-\left(1-\delta \right)\times \left(V_{\text{half}}-V\right)}{\sigma }\right)$$

where:

V — the membrane potential in mV

$V_{\text{half}}$ —alhe half-activation potential in mV

σ — the inverse slope in mV

δ — the skew parameter of the time constant curve (unitless)

K — the maximum rate parameter in ms−1

$\tau_{0}$ — the rate-limiting factor (minimum time constant) in ms

For each state variable of a channel, we fit the set of 5 parameters of the system of equations (4 – 7), namely $V_{\text{half}},\;\sigma ,\;K,\;\delta$, and $\tau_{0}$, to the data in the form of activation (inactivation) curves derived from the original mod files for membrane potentials in the range from − 100 to 100 mV . The fitting process is implemented in the symfit Python library (https://symfit.readthedocs.io) to fit both curves for the steady state and the time constant simultaneously. The temperature is set to the original temperature before getting data to fit. We noticed that, while accurate for both curves, simultaneous fitting results in significant changes in the voltage and current dynamics. Therefore, we introduced a second additional fit for the steady state alone, sacrificing fitting accuracy for the time constant but preserving voltage and current dynamics. Finally, a new .mod file is created from a JINJA (https://jinja.palletsprojects.com) template and is immediately available to replace the original mechanism in the neuronal model.

### 4.4 Morphology reduction

We extended the analytical impedance-based neuron reduce approach proposed by Amsalem et al., 2020 [Amsalem et al., 2020] and integrated it into our GUI. The original neuron_reduce algorithm maps a dendritic subtree to a single cylinder with both ends sealed, preserving:

- specific membrane resistivity, $R_{m}$ in $Ohm\times {\text{cm}}^{2}$
- specific membrane capacitance, $C_{m}$ in $F/{\text{cm}}^{2}$
- specific axial resistivity, $R_{a}$ in Ohm×cm
- the transfer impedance from the electrotonically most distal dendritic tip to the soma, $\left|Z_{0,L}\left(\omega \right)\right|$
- the input resistance at the soma end (when disconnected from the soma), $\left|Z_{0,0}\left(\omega \right)\right|$

Equations (1)–(11) in the original paper describe the unique cylindrical cable (with a specific diameter, d and length, L, and the given membrane and axial properties) that preserves the values of $\left|Z_{0,L}\left(\omega \right)\right|$ and $\left|Z_{0,0}\left(\omega \right)\right|$. In the original implementation, the entire subtree of each stem dendrite (e.g., the entire apical subtree) is mapped to a single corresponding cylinder. We extended this approach to allow a user to select any section they want and map the inclusive subtree of this section (including the section itself) to a single cylinder. When the user selects the desired section using the GUI and clicks the button ‘Reduce subtree’ the inclusive subtree is disconnected from the cell and parameters for its equivalent cylinder are calculated. The exclusive subtree of the section is then removed, and the section’s length and diameter are updated with the new calculated values before reconnecting it to its original parent. As in the original method, the reduced model is compartmentalized into segments (typically with a spatial resolution of 0.1 λ ), and channel conductances are adjusted according to the mapping between the original and the reduced segments. In order to introduce a more general workflow, where synapses can be allocated on the already reduced model, we removed the step from the original algorithm that mapped synapses to the corresponding cylinder.

### 4.5 Validation

We utilized a semi-automated approach for model validation. The user manually specifies the simulation parameters required for a specific validation protocol using the GUI (Table 1). Depending on the protocol, one or multiple simulation runs are performed in NEURON [Hines, 2009], and the simulated voltage values are used for further calculations. Input resistance is calculated according to the formula: $R_{\text{in}}=\frac{V_{\text{offset}}-V_{\text{onset}}}{I_{\text{ext}}}$. The membrane time constant (τ) is derived by fitting an exponential equation to the decaying part of the voltage curve after the stimulus onset. Voltage attenuation is calculated for a user-specified set of segments by measuring the voltage response at different points along the dendrite. For each segment, the voltage attenuation is computed as the ratio of the voltage change at the segment $\left(\Delta V_{\text{seg}}\right)$ to the voltage change at the stimulation site $\left(\Delta V_{\text{stim}}\right)$. The distances from the soma to each segment are also recorded, and the attenuation is plotted against these distances. For detecting somatic action potentials and measuring their amplitude and half-width, we used the SciPy [Virtanen et al., 2020] Python library for peak detection in a signal. This involves identifying the peaks in the voltage trace and calculating the time difference between the points where the voltage is half of the peak amplitude. The somatic f–I curve is built by injecting a range of current amplitudes (0 to 1000 pA in steps of 100 pA) into the soma and recording the number of action potentials generated at each current level. The firing rate is plotted as a function of the injected current amplitude. Dendritic nonlinearities are derived by measuring the voltage response in a dendrite to increasing synaptic input weights of a single synapse. The unitary voltage response (EPSP) is determined, and the actual voltage responses for increasing synaptic weights are compared to the expected linear sum of unitary responses. The sag ratio is calculated according to the formula: Sag $\text{ratio}=\frac{a}{b}$, where $a=V_{\text{offset}}-V_{\text{min}}$ and $b=V_{\text{onset}}-V_{\text{min}}$. This ratio is derived from the voltage response to a hyperpolarizing current injection in the presence of HCN channels (h current), with $V_{\text{onset}}$ being the initial voltage before the injection, $V_{\text{min}}$ the minimum voltage reached, and $V_{\text{offset}}$ the voltage at the end of the injection.

## Figures and Tables

**Table 1:** Validation protocols

Validation protocol	Readout	Necessary membrane parameters	Recordings	Stimuli
Input resistance	$R_{\text{in}},\;\text{M}\Omega$	Passive	Any single segment	Negative step current injection
Membrane time constant	τ,ms	Passive	Any single segment	Negative step current injection
Rheobase current	$I_{\text{rh}},\;\text{pA}$	At least Na and Kdr	Soma	Positive step current injection
Somatic spikes	AP times, amplitude, half-width, rate, ISI	At least Na and Kdr	Soma	Positive step current injection above the rheobase value
Somatic f/I curve	$\text{Rate}\left(I_{\text{ext}}\right),\;Hz$	At least Na and Kdr	Soma	Positive step current injection (amplitude automatically increased)
Dendritic nonlinearity	EPSP, mV	Dendritic ion channels	Dendritic segment	Excitatory synapse (weight automatically increased)
Voltage attenuation	$\Delta V_{0}/\Delta V_{i},\;\text{unitless}$	Passive	At least two segments	Negative step current injection
Sag ratio	$\frac{V_{\text{end}}-V_{\text{min}}}{V_{\text{start}}-V_{\text{min}}}$	HCN channels (h current)	Any single segment	Negative step current injection

## References

[R1] Rall Wilfrid. Branching dendritic trees and motoneuron membrane resistivity. Experimental Neurology, 1(5):491–527, November 1959. ISSN 00144886. doi: 10.1016/0014-4886(59)90046-9. URL https://linkinghub.elsevier.com/retrieve/pii/0014488659900469. 14435979

[R2] Spencer W. A. and Kandel E. R.. ELECTROPHYSIOLOGY OF HIPPOCAMPAL NEURONS: IV. FAST PREPOTENTIALS. Journal of Neurophysiology, 24(3):272–285, May 1961. ISSN 0022–3077, 1522–1598. doi: 10.1152/jn.1961.24.3.272. URL https://www.physiology.org/doi/10.1152/jn.1961.24.3.272. 25286477

[R3] Golding Nace L and Spruston Nelson. Dendritic Sodium Spikes Are Variable Triggers of Axonal Action Potentials in Hippocampal CA1 Pyramidal Neurons. Neuron, 21(5):1189–1200, November 1998. ISSN 08966273. doi: 10.1016/S0896-6273(00)80635-2. URL https://linkinghub.elsevier.com/retrieve/pii/S0896627300806352. 9856473

[R4] Schiller Jackie and Schiller Yitzhak. NMDA receptor-mediated dendritic spikes and coincident signal amplification. Current Opinion in Neurobiology, 11(3):343–348, June 2001. ISSN 09594388. doi: 10.1016/S0959-4388(00)00217-8. URL https://linkinghub.elsevier.com/retrieve/pii/S0959438800002178. 11399433

[R5] Llinas R and Nicholson C. Electrophysiological properties of dendrites and somata in alligator Purkinje cells. Journal of Neurophysiology, 34(4):532–551, July 1971. ISSN 0022–3077, 1522–1598. doi: 10.1152/jn.1971.34.4.532. URL https://www.physiology.org/doi/10.1152/jn.1971.34.4.532. 4329778

[R6] Llinás R and Hess R. Tetrodotoxin-resistant dendritic spikes in avian Purkinje cells. Proceedings of the National Academy of Sciences, 73(7):2520–2523, July 1976. ISSN 0027–8424, 1091–6490. doi: 10.1073/pnas.73.7.2520. URL https://pnas.org/doi/full/10.1073/pnas.73.7.2520. PMC430632 1065905

[R7] Hines M. L. and Carnevale N. T.. Neuron: A Tool for Neuroscientists. The Neuroscientist, 7 (2):123–135, April 2001. ISSN 1073–8584, 1089–4098. doi: 10.1177/107385840100700207. URL http://journals.sagepub.com/doi/10.1177/107385840100700207. 11496923

[R8] Bokeh Development Team. Bokeh: Python library for interactive visualization, 2014. URL http://www.bokeh.pydata.org.

[R9] Ascoli Giorgio A., Donohue Duncan E., and Halavi Maryam. NeuroMorpho.Org: A Central Resource for Neuronal Morphologies. The Journal of Neuroscience, 27(35):9247–9251, August 2007. ISSN 0270–6474, 1529–2401. doi: 10.1523/JNEUROSCI.2055-07.2007. URL https://www.jneurosci.org/lookup/doi/10.1523/JNEUROSCI.2055-07.2007. 17728438 PMC6673130

[R10] Mehta Ketan, Ljungquist Bengt, Ogden James, Nanda Sumit, Ascoli Ruben G., Ng Lydia, and Ascoli Giorgio A.. Online conversion of reconstructed neural morphologies into standardized SWC format. Nature Communications, 14(1):7429, November 2023. ISSN 2041–1723. doi: 10.1038/s41467-023-42931-x. URL https://www.nature.com/articles/s41467-023-42931-x. PMC10654402 37973857

[R11] Park Jiyoung, Papoutsi Athanasia, Ash Ryan T., Marin Miguel A., Poirazi Panayiota, and Smirnakis Stelios M.. Contribution of apical and basal dendrites to orientation encoding in mouse V1 L2/3 pyramidal neurons. Nature Communications, 10(1):5372, November 2019. ISSN 2041–1723. doi: 10.1038/s41467-019-13029-0. URL https://www.nature.com/articles/s41467-019-13029-0. PMC6879601 31772192

[R12] Carnevale Nicholas T. and Hines Michael L.. The NEURON Book. Cambridge University Press, 1 edition, January 2006. ISBN 978–0-521–84321-8 978–0-521–11563-6 978–0-511–54161-2. doi: 10.1017/CBO9780511541612. URL https://www.cambridge.org/core/product/identifier/9780511541612/type/book.

[R13] Korogod Sergiy M., Stern Javier E., and Cymbalyuk Gennady S.. Microgeometrical dendritic factors predict electrical decoupling between somatic and dendritic compartments in magnocellular neurosecretory neurons. Frontiers in Cellular Neuroscience, 17:1125029, March 2023. ISSN 1662–5102. doi: 10.3389/fncel.2023.1125029. URL https://www.frontiersin.org/articles/10.3389/fncel.2023.1125029/full. PMC10081025 37032839

[R14] Hodgkin A. L. and Huxley A. F.. A quantitative description of membrane current and its application to conduction and excitation in nerve. The Journal of Physiology, 117(4):500–544, August 1952. ISSN 0022–3751, 1469–7793. doi: 10.1113/jphysiol.1952.sp004764. URL https://physoc.onlinelibrary.wiley.com/doi/10.1113/jphysiol.1952.sp004764. 12991237 PMC1392413

[R15] Petousakis Konstantinos-Evangelos, Apostolopoulou Anthi A., and Poirazi Panayiota. The impact of Hodgkin-Huxley models on dendritic research. The Journal of Physiology, 601(15):3091–3102, August 2023a. ISSN 0022–3751, 1469–7793. doi: 10.1113/JP282756. URL https://physoc.onlinelibrary.wiley.com/doi/10.1113/JP282756. 36218068 PMC10600871

[R16] Hines M. L. and Carnevale N. T.. Expanding NEURON’s Repertoire of Mechanisms with NMODL. Neural Computation, 12(5):995–1007, May 2000. ISSN 0899–7667, 1530–888X. doi: 10.1162/089976600300015475. URL https://direct.mit.edu/neco/article/12/5/995-1007/6367. 10905805

[R17] Gleeson Padraig, Crook Sharon, Cannon Robert C., Hines Michael L., Billings Guy O., Farinella Matteo, Morse Thomas M., Davison Andrew P., Ray Subhasis, Bhalla Upinder S., Barnes Simon R., Dimitrova Yoana D., and Silver R. Angus. NeuroML: A Language for Describing Data Driven Models of Neurons and Networks with a High Degree of Biological Detail. PLoS Computational Biology, 6(6):e1000815, June 2010. ISSN 1553–7358. doi: 10.1371/journal.pcbi.1000815. URL https://dx.plos.org/10.1371/journal.pcbi.1000815. PMC2887454 20585541

[R18] Podlaski William F, Seeholzer Alexander, Groschner Lukas N, Miesenböck Gero, Ranjan Rajnish, and Vogels Tim P. Mapping the function of neuronal ion channels in model and experiment. eLife, 6:e22152, March 2017. ISSN 2050–084X. doi: 10.7554/eLife.22152. URL https://elifesciences.org/articles/22152. PMC5340531 28267430

[R19] Kumbhar Pramod, Awile Omar, Keegan Liam, Alonso Jorge Blanco, King James, Hines Michael, and Schürmann Felix. An Optimizing Multi-platform Source-to-source Compiler Framework for the NEURON MODeling Language. In Computational Science - ICCS 2020, volume 12137, pages 45–58. Springer International Publishing, Cham, 2020. ISBN 978–3-030–50370-3 978–3-030–50371-0. doi: 10.1007/978-3-030-50371-0_4. URL http://link.springer.com/10.1007/978-3-030-50371-0_4. Series Title: Lecture Notes in Computer Science.

[R20] Ranjan Rajnish, Khazen Georges, Gambazzi Luca, Ramaswamy Srikanth, Hill Sean L., Felix Schürmann, and Henry Markram. Channelpedia: An Integrative and Interactive Database for Ion Channels. Frontiers in Neuroinformatics, 5, 2011. ISSN 1662–5196. doi: 10.3389/fninf.2011.00036. URL http://journal.frontiersin.org/article/10.3389/fninf.2011.00036/abstract. PMC3248699 22232598

[R21] Ranjan Rajnish, Logette Emmanuelle, Dorp Stijn V., Kalaimaken Hervé A., Herzog Mirjia, Joffraud Magali S.E., Scantamburlo Enrico, Johnston Katherine G., Journe Adrien, and Markram Henry. Channelome: A comprehensive resource for voltage-gated ion channel kinetics. Biophysical Journal, 123(3):527a, February 2024. ISSN 00063495. doi: 10.1016/j.bpj.2023.11.3186. URL https://linkinghub.elsevier.com/retrieve/pii/S0006349523038870.38258291

[R22] Johnston Daniel and Narayanan Rishikesh. Active dendrites: colorful wings of the mysterious butterflies. Trends in Neurosciences, 31(6):309–316, June 2008. ISSN 01662236. doi: 10.1016/j.tins.2008.03.004. URL https://linkinghub.elsevier.com/retrieve/pii/S0166223608001197. 18471907

[R23] Yu Frank H., Yarov-Yarovoy Vladimir, Gutman George A., and Catterall William A.. Overview of Molecular Relationships in the Voltage-Gated Ion Channel Superfamily. Pharmacological Reviews, 57(4):387–395, December 2005. ISSN 0031–6997, 1521–0081. doi: 10.1124/pr.57.4.13. URL http://pharmrev.aspetjournals.org/lookup/doi/10.1124/pr.57.4.13. 16382097

[R24] Reuveni I, Friedman A, Amitai Y, and Gutnick Mj. Stepwise repolarization from Ca2+ plateaus in neocortical pyramidal cells: evidence for nonhomogeneous distribution of HVA Ca2+ channels in dendrites. The Journal of Neuroscience, 13(11):4609–4621, November 1993. ISSN 0270–6474, 1529–2401. doi: 10.1523/JNEUROSCI.13-11-04609.1993. URL https://www.jneurosci.org/lookup/doi/10.1523/JNEUROSCI.13-11-04609.1993. 8229187 PMC6576337

[R25] Westenbroek Ruth E., Ahlijanian Michael K., and Catterall William A.. Clustering of L-type Ca2+ channels at the base of major dendrites in hippocampal pyramidal neurons. Nature, 347 (6290):281–284, September 1990. ISSN 0028–0836, 1476–4687. doi: 10.1038/347281a0. URL https://www.nature.com/articles/347281a0. 2169591

[R26] Magee J C and Johnston D. Characterization of single voltage-gated Na+ and Ca2+ channels in apical dendrites of rat CA1 pyramidal neurons. The Journal of Physiology, 487(1):67–90, August 1995. ISSN 0022–3751, 1469–7793. doi: 10.1113/jphysiol.1995.sp020862. URL https://physoc.onlinelibrary.wiley.com/doi/10.1113/jphysiol.1995.sp020862. 7473260 PMC1156600

[R27] Magee J. C., Christofi G., Miyakawa H., Christie B., Lasser-Ross N., and Johnston D.. Sub-threshold synaptic activation of voltage-gated Ca2+ channels mediates a localized Ca2+ in-flux into the dendrites of hippocampal pyramidal neurons. Journal of Neurophysiology, 74(3): 1335–1342, September 1995. ISSN 0022–3077, 1522–1598. doi: 10.1152/jn.1995.74.3.1335. URL https://www.physiology.org/doi/10.1152/jn.1995.74.3.1335. 7500154

[R28] Yuste Rafael, Gutnick Michael J., Saar Drorit, Delaney Kerry R., and Tank David W.. Ca2+ accumulations in dendrites of neocortical pyramidal neurons: An apical band and evidence for two functional compartments. Neuron, 13(1):23–43, July 1994. ISSN 08966273. doi: 10.1016/0896-6273(94)90457-X. URL https://linkinghub.elsevier.com/retrieve/pii/089662739490457X. 8043278

[R29] Magee Jeffrey C.. Dendritic Hyperpolarization-Activated Currents Modify the Integrative Properties of Hippocampal CA1 Pyramidal Neurons. The Journal of Neuroscience, 18(19):7613–7624, October 1998. ISSN 0270–6474, 1529–2401. doi: 10.1523/JNEUROSCI.18-19-07613.1998. URL https://www.jneurosci.org/lookup/doi/10.1523/JNEUROSCI.18-19-07613.1998. 9742133 PMC6793032

[R30] Lörincz Andrea, Notomi Takuya, Tamás Gábor, Shigemoto Ryuichi, and Nusser Zoltan. Polarized and compartment-dependent distribution of HCN1 in pyramidal cell dendrites. Nature Neuroscience, 5(11):1185–1193, November 2002. ISSN 1097–6256, 1546–1726. doi: 10.1038/nn962. URL https://www.nature.com/articles/nn962. 12389030

[R31] Kole Maarten H. P., Hallermann Stefan, and Stuart Greg J.. Single *I*_h_ Channels in Pyramidal Neuron Dendrites: Properties, Distribution, and Impact on Action Potential Output. The Journal of Neuroscience, 26(6):1677–1687, February 2006. ISSN 0270–6474, 1529–2401. doi: 10.1523/JNEUROSCI.3664-05.2006. URL https://www.jneurosci.org/lookup/doi/10.1523/JNEUROSCI.3664-05.2006. 16467515 PMC6793638

[R32] Stuart Greg and Spruston Nelson. Determinants of Voltage Attenuation in Neocortical Pyramidal Neuron Dendrites. The Journal of Neuroscience, 18(10):3501–3510, May 1998. ISSN 0270–6474, 1529–2401. doi: 10.1523/JNEUROSCI.18-10-03501.1998. URL https://www.jneurosci.org/lookup/doi/10.1523/JNEUROSCI.18-10-03501.1998. 9570781 PMC6793161

[R33] Migliore Michele and Shepherd Gordon M.. Emerging rules for the distributions of active dendritic conductances. Nature Reviews Neuroscience, 3(5):362–370, May 2002. ISSN 1471–003X, 1471–0048. doi: 10.1038/nrn810. URL https://www.nature.com/articles/nrn810. 11988775

[R34] Nusser Zoltan. Variability in the subcellular distribution of ion channels increases neuronal diversity. Trends in Neurosciences, 32(5):267–274, May 2009. ISSN 01662236. doi: 10.1016/j.tins.2009.01.003. URL https://linkinghub.elsevier.com/retrieve/pii/S0166223609000423. 19299025

[R35] Shah Mala M., Hammond Rebecca S., and Hoffman Dax A.. Dendritic ion channel trafficking and plasticity. Trends in Neurosciences, 33(7):307–316, July 2010. ISSN 01662236. doi: 10.1016/j.tins.2010.03.002. URL https://linkinghub.elsevier.com/retrieve/pii/S0166223610000366. 20363038 PMC2902701

[R36] Poirazi Panayiota, Brannon Terrence, and Mel Bartlett W.. Pyramidal Neuron as Two-Layer Neural Network. Neuron, 37(6):989–999, March 2003. ISSN 08966273. doi: 10.1016/S0896-6273(03)00149-1. URL https://linkinghub.elsevier.com/retrieve/pii/S0896627303001491. 12670427

[R37] Hay Etay, Hill Sean, Schürmann Felix, Markram Henry, and Segev Idan. Models of Neocortical Layer 5b Pyramidal Cells Capturing a Wide Range of Dendritic and Perisomatic Active Properties. PLoS Computational Biology, 7(7):e1002107, July 2011. ISSN 1553–7358. doi: 10.1371/journal.pcbi.1002107. URL https://dx.plos.org/10.1371/journal.pcbi.1002107. PMC3145650 21829333

[R38] Polsky Alon, Mel Bartlett W, and Schiller Jackie. Computational subunits in thin dendrites of pyramidal cells. Nature Neuroscience, 7(6):621–627, June 2004. ISSN 1097–6256, 1546–1726. doi: 10.1038/nn1253. URL https://www.nature.com/articles/nn1253. 15156147

[R39] Losonczy Attila and Magee Jeffrey C.. Integrative Properties of Radial Oblique Dendrites in Hippocampal CA1 Pyramidal Neurons. Neuron, 50(2):291–307, April 2006. ISSN 08966273. doi: 10.1016/j.neuron.2006.03.016. URL https://linkinghub.elsevier.com/retrieve/pii/S0896627306002133. 16630839

[R40] Takahashi Naoya, Kitamura Kazuo, Matsuo Naoki, Mayford Mark, Kano Masanobu, Matsuki Norio, and Ikegaya Yuji. Locally Synchronized Synaptic Inputs. Science, 335(6066):353–356, January 2012. ISSN 0036–8075, 1095–9203. doi: 10.1126/science.1210362. URL https://www.science.org/doi/10.1126/science.1210362. 22267814

[R41] Tran-Van-Minh Alexandra, Cazé Romain D., Abrahamsson Therése, Cathala Laurence, Gutkin Boris S., and DiGregorio David A.. Contribution of sublinear and supralinear dendritic integration to neuronal computations. Frontiers in Cellular Neuroscience, 9, March 2015. ISSN 1662–5102. doi: 10.3389/fncel.2015.00067. URL http://journal.frontiersin.org/Article/10.3389/fncel.2015.00067/abstract. PMC4371705 25852470

[R42] Doron Michael, Chindemi Giuseppe, Muller Eilif, Markram Henry, and Segev Idan. Timed Synaptic Inhibition Shapes NMDA Spikes, Influencing Local Dendritic Processing and Global I/O Properties of Cortical Neurons. Cell Reports, 21(6):1550–1561, November 2017. ISSN 22111247. doi: 10.1016/j.celrep.2017.10.035. URL https://linkinghub.elsevier.com/retrieve/pii/S2211124717314675. 29117560

[R43] Du Kai, Wu Yu-Wei, Lindroos Robert, Liu Yu, Rózsa Balázs, Katona Gergely, Ding Jun B., and Kotaleski Jeanette Hellgren. Cell-type-specific inhibition of the dendritic plateau potential in striatal spiny projection neurons. Proceedings of the National Academy of Sciences, 114(36), September 2017. ISSN 0027–8424, 1091–6490. doi: 10.1073/pnas.1704893114. URL https://pnas.org/doi/full/10.1073/pnas.1704893114. PMC5594658 28827326

[R44] Markram Henry, Toledo-Rodriguez Maria, Wang Yun, Gupta Anirudh, Silberberg Gilad, and Wu Caizhi. Interneurons of the neocortical inhibitory system. Nature Reviews Neuroscience, 5(10):793–807, October 2004. ISSN 1471–003X, 1471–0048. doi: 10.1038/nrn1519. URL https://www.nature.com/articles/nrn1519. 15378039

[R45] Van Versendaal Daniëlle and Levelt Christiaan N.. Inhibitory interneurons in visual cortical plasticity. Cellular and Molecular Life Sciences, 73(19):3677–3691, October 2016. ISSN 1420–682X, 1420–9071. doi: 10.1007/s00018-016-2264-4. URL http://link.springer.com/10.1007/s00018-016-2264-4. 27193323 PMC5002041

[R46] Amsalem Oren, Eyal Guy, Rogozinski Noa, Gevaert Michael, Kumbhar Pramod, Schürmann Felix, and Segev Idan. An efficient analytical reduction of detailed nonlinear neuron models. Nature Communications, 11(1):288, January 2020. ISSN 2041–1723. doi: 10.1038/s41467-019-13932-6. URL https://www.nature.com/articles/s41467-019-13932-6. PMC6962154 31941884

[R47] Mel Bartlett W.. Information Processing in Dendritic Trees. Neural Computation, 6(6):1031–1085, November 1994. ISSN 0899–7667, 1530–888X. doi: 10.1162/neco.1994.6.6.1031. URL https://direct.mit.edu/neco/article/6/6/1031-1085/5835.

[R48] Häusser Michael and Mel Bartlett. Dendrites: bug or feature? Current Opinion in Neurobiology, 13(3):372–383, June 2003. ISSN 09594388. doi: 10.1016/S0959-4388(03)00075-8. URL https://linkinghub.elsevier.com/retrieve/pii/S0959438803000758. 12850223

[R49] Poirazi Panayiota and Papoutsi Athanasia. Illuminating dendritic function with computational models. Nature Reviews Neuroscience, 21(6):303–321, June 2020. ISSN 1471–003X, 1471–0048. doi: 10.1038/s41583-020-0301-7. URL https://www.nature.com/articles/s41583-020-0301-7. 32393820

[R50] Larkum Matthew E., Wu Jiameng, Duverdin Sarah A., and Gidon Albert. The Guide to Dendritic Spikes of the Mammalian Cortex In Vitro and In Vivo. Neuroscience, 489:15–33, May 2022. ISSN 03064522. doi: 10.1016/j.neuroscience.2022.02.009. URL https://linkinghub.elsevier.com/retrieve/pii/S030645222200063X. 35182699

[R51] Markram Henry, Muller Eilif, Ramaswamy Srikanth, and Reconstruction and Simulation of Neocortical Microcircuitry. Cell, 163(2):456–492, October 2015. ISSN 00928674. doi: 10.1016/j.cell.2015.09.029. URL https://linkinghub.elsevier.com/retrieve/pii/S0092867415011915. 26451489

[R52] Billeh Yazan N., Cai Binghuang, Gratiy Sergey L., Dai Kael, Iyer Ramakrishnan, Gouwens Nathan W., Abbasi-Asl Reza, Jia Xiaoxuan, Siegle Joshua H., Olsen Shawn R., Koch Christof, Mihalas Stefan, and Arkhipov Anton. Systematic Integration of Structural and Functional Data into Multi-scale Models of Mouse Primary Visual Cortex. Neuron, 106(3):388–403.e18, May 2020. ISSN 08966273. doi: 10.1016/j.neuron.2020.01.040. URL https://linkinghub.elsevier.com/retrieve/pii/S0896627320300672. 32142648

[R53] Stimberg Marcel, Brette Romain, and Goodman Dan Fm. Brian 2, an intuitive and efficient neural simulator. eLife, 8:e47314, August 2019. ISSN 2050–084X. doi: 10.7554/eLife.47314. URL https://elifesciences.org/articles/47314. PMC6786860 31429824

[R54] Feng Linqing, Zhao Ting, and Kim Jinhyun. neuTube 1.0: A New Design for Efficient Neuron Reconstruction Software Based on the SWC Format. eneuro, 2(1):ENEURO.0049–14.2014, January 2015. ISSN 2373–2822. doi: 10.1523/ENEURO.0049-14.2014. URL https://www.eneuro.org/lookup/doi/10.1523/ENEURO.0049-14.2014. PMC4586918 26464967

[R55] Bozelos Panagiotis, Stefanou Stefanos S., Bouloukakis Georgios, Melachrinos Constantinos, and Poirazi Panayiota. REMOD: A Tool for Analyzing and Remodeling the Dendritic Architecture of Neural Cells. Frontiers in Neuroanatomy, 9, January 2016. ISSN 1662–5129. doi: 10.3389/fnana.2015.00156. URL http://journal.frontiersin.org/Article/10.3389/fnana.2015.00156/abstract. PMC4701929 26778971

[R56] Bakker R., García-Amado M., Evangelio M., Clascá F., and Tiesinga P.. P271 workflow, data format and tools to register neuron morphologies to a reference brain atlas. In Newton A. J. H., Seidenstein A. H., McDougal R. A., , editors, 26th Annual Computational Neuroscience Meeting (CNS*2017): Part 3, volume 18, Suppl 1 of BMC Neuroscience, page 60, 2017. doi: 10.1186/s12868-017-0372-1. URL https://bmcneurosci.biomedcentral.com/articles/10.1186/s12868-017-0372-1.

[R57] Maraver Jose Juan Aliaga, Mata Susana, Benavides-Piccione Ruth, DeFelipe Javier, and Pastor Luis. A Method for the Symbolic Representation of Neurons. Frontiers in Neuroanatomy, 12:106, December 2018. ISSN 1662–5129. doi: 10.3389/fnana.2018.00106. URL https://www.frontiersin.org/article/10.3389/fnana.2018.00106/full. 30618651 PMC6305400

[R58] Cuntz Hermann, Forstner Friedrich, Borst Alexander, and Häusser Michael. The TREES Tool-box—Probing the Basis of Axonal and Dendritic Branching. Neuroinformatics, 9(1):91–96, March 2011. ISSN 1539–2791, 1559–0089. doi: 10.1007/s12021-010-9093-7. URL http://link.springer.com/10.1007/s12021-010-9093-7. 21222051 PMC7612393

[R59] Brito Juan P., Mata Susana, Bayona Sofia, Pastor Luis, DeFelipe Javier, and Benavides-Piccione Ruth. Neuronize: a tool for building realistic neuronal cell morphologies. Frontiers in Neuroanatomy, 7, 2013. ISSN 1662–5129. doi: 10.3389/fnana.2013.00015. URL http://journal.frontiersin.org/article/10.3389/fnana.2013.00015/abstract. PMC3669758 23761740

[R60] Velasco Ivan, Garcia-Cantero Juan J., Brito Juan P., Bayona Sofia, Pastor Luis, and Mata Susana. NeuroEditor: a tool to edit and visualize neuronal morphologies. Frontiers in Neuroanatomy, 18:1342762, February 2024. ISSN 1662–5129. doi: 10.3389/fnana.2024.1342762. URL https://www.frontiersin.org/articles/10.3389/fnana.2024.1342762/full. PMC10902916 38425804

[R61] Koene Randal A., Tijms Betty, Van Hees Peter, Postma Frank, De Ridder Alexander, Ramakers Ger J. A., Van Pelt Jaap, and Van Ooyen Arjen. NETMORPH: A Framework for the Stochastic Generation of Large Scale Neuronal Networks With Realistic Neuron Morphologies. Neuroinformatics, 7(3):195–210, September 2009. ISSN 1539–2791, 1559–0089. doi: 10.1007/s12021-009-9052-3. URL http://link.springer.com/10.1007/s12021-009-9052-3. 19672726

[R62] Van Geit Werner, Gevaert Michael, Chindemi Giuseppe, Rössert Christian, Courcol Jean-Denis, Muller Eilif B., Schürmann Felix, Segev Idan, and Markram Henry. BluePyOpt: Leveraging Open Source Software and Cloud Infrastructure to Optimise Model Parameters in Neuroscience. Frontiers in Neuroinformatics, 10, June 2016. ISSN 1662–5196. doi: 10.3389/fninf.2016.00017. URL http://journal.frontiersin.org/Article/10.3389/fninf.2016.00017/abstract. PMC4896051 27375471

[R63] Gonçalves Pedro J, Lueckmann Jan-Matthis, Deistler Michael, Nonnenmacher Marcel, Öcal Kaan, Bassetto Giacomo, Chintaluri Chaitanya, Podlaski William F, Haddad Sara A, Vogels Tim P, Greenberg David S, and Macke Jakob H. Training deep neural density estimators to identify mechanistic models of neural dynamics. eLife, 9:e56261, September 2020. ISSN 2050–084X. doi: 10.7554/eLife.56261. URL https://elifesciences.org/articles/56261. PMC7581433 32940606

[R64] Jones Ilenna Simone and Kording Konrad Paul. Efficient optimization of ODE neuron models using gradient descent, 2024. URL https://arxiv.org/abs/2407.04025. Version Number: 1.

[R65] McDougal Robert A., Morse Thomas M., Carnevale Ted, Marenco Luis, Wang Rixin, Migliore Michele, Miller Perry L., Shepherd Gordon M., and Hines Michael L. Twenty years of ModelDB and beyond: building essential modeling tools for the future of neuroscience. Journal of Computational Neuroscience, 42(1):1–10, February 2017. ISSN 0929–5313, 1573–6873. doi: 10.1007/s10827-016-0623-7. URL http://link.springer.com/10.1007/s10827-016-0623-7. 27629590 PMC5279891

[R66] Dai Kael, Hernando Juan, Billeh Yazan N., Gratiy Sergey L., Planas Judit, Davison Andrew P., Dura-Bernal Salvador, Gleeson Padraig, Devresse Adrien, Dichter Benjamin K., Gevaert Michael, King James G., Van Geit Werner A. H., Povolotsky Arseny V., Muller Eilif, Courcol Jean-Denis, and Arkhipov Anton. The SONATA data format for efficient description of large-scale network models. PLOS Computational Biology, 16(2):e1007696, February 2020. ISSN 1553–7358. doi: 10.1371/journal.pcbi.1007696. URL https://dx.plos.org/10.1371/journal.pcbi.1007696. PMC7058350 32092054

[R67] the INCF Multiscale Modeling Taskforce and Anatoli Gorchetchnikov. NineML - a description language for spiking neuron network modeling: the user layer. BMC Neuroscience, 11(S1):P71, 1471–2202-11-S1-P71, July 2010. ISSN 1471–2202. doi: 10.1186/1471-2202-11-S1-P71. URL https://bmcneurosci.biomedcentral.com/articles/10.1186/1471-2202-11-S1-P71.

[R68] Reva Maria, Rössert Christian, Arnaudon Alexis, Damart Tanguy, Mandge Darshan, Tuncel Anıl, Ramaswamy Srikanth, Markram Henry, and Van Geit Werner. A universal workflow for creation, validation, and generalization of detailed neuronal models. Patterns, 4(11):100855, November 2023. ISSN 26663899. doi: 10.1016/j.patter.2023.100855. URL https://linkinghub.elsevier.com/retrieve/pii/S2666389923002398. PMC10682753 38035193

[R69] Dura-Bernal Salvador, Suter Benjamin A, Gleeson Padraig, Cantarelli Matteo, Quintana Adrian, Rodriguez Facundo, Kedziora David J, Chadderdon George L, Kerr Cliff C, Neymotin Samuel A, McDougal Robert A, Hines Michael, Shepherd Gordon Mg, and Lytton William W. NetPyNE, a tool for data-driven multiscale modeling of brain circuits. eLife, 8:e44494, April 2019. ISSN 2050–084X. doi: 10.7554/eLife.44494. URL https://elifesciences.org/articles/44494. PMC6534378 31025934

[R70] Stratford K., Mason A., Larkman A., Major G., and Jack J.. The modeling of pyramidal neurons in the visual cortex. In Durbin R., Miall C., and Mitchison G., editors, The Computing Neuron, pages 296–321. Addison-Wesley, London, 1989.

[R71] Bush Paul C. and Sejnowski Terrence J.. Reduced compartmental models of neocortical pyramidal cells. Journal of Neuroscience Methods, 46(2):159–166, February 1993. ISSN 01650270. doi: 10.1016/0165-0270(93)90151-G. URL https://linkinghub.elsevier.com/retrieve/pii/016502709390151G. 8474259

[R72] Destexhe Alain. Simplified models of neocortical pyramidal cells preserving somatodendritic voltage attenuation. Neurocomputing, 38–40:167–173, June 2001. ISSN 09252312. doi: 10.1016/S0925-2312(01)00428-3. URL https://linkinghub.elsevier.com/retrieve/pii/S0925231201004283.

[R73] Hendrickson Eric B., Edgerton Jeremy R., and Jaeger Dieter. The capabilities and limitations of conductance-based compartmental neuron models with reduced branched or un-branched morphologies and active dendrites. Journal of Computational Neuroscience, 30(2): 301–321, April 2011. ISSN 0929–5313, 1573–6873. doi: 10.1007/s10827-010-0258-z. URL http://link.springer.com/10.1007/s10827-010-0258-z. 20623167 PMC3058356

[R74] Marasco Addolorata, Limongiello Alessandro, and Migliore Michele. Using Strahler’s analysis to reduce up to 200-fold the run time of realistic neuron models. Scientific Reports, 3(1):2934, October 2013. ISSN 2045–2322. doi: 10.1038/srep02934. URL https://www.nature.com/articles/srep02934. 24121727 PMC3796311

[R75] Wybo Willem Am, Jordan Jakob, Ellenberger Benjamin, Mengual Ulisses Marti, Nevian Thomas, and Senn Walter. Data-driven reduction of dendritic morphologies with preserved dendro-somatic responses. eLife, 10:e60936, January 2021. ISSN 2050–084X. doi: 10.7554/eLife.60936. URL https://elifesciences.org/articles/60936. PMC7837682 33494860

[R76] Otor Yara, Achvat Shay, Cermak Nathan, Benisty Hadas, Abboud Maisan, Barak Omri, Schiller Yitzhak, Alon Poleg-Polsky, and Jackie Schiller. Dynamic compartmental computations in tuft dendrites of layer 5 neurons during motor behavior. Science, 376(6590):267–275, April 2022. ISSN 0036–8075, 1095–9203. doi: 10.1126/science.abn1421. URL https://www.science.org/doi/10.1126/science.abn1421. 35420959

[R77] Francioni Valerio and Harnett Mark T.. Rethinking Single Neuron Electrical Compartmentalization: Dendritic Contributions to Network Computation In Vivo. Neuroscience, 489:185–199, May 2022. ISSN 03064522. doi: 10.1016/j.neuroscience.2021.05.038. URL https://linkinghub.elsevier.com/retrieve/pii/S0306452221002852. 34116137

[R78] Stuyt George, Godenzini Luca, and Palmer Lucy M.. Local and Global Dynamics of Dendritic Activity in the Pyramidal Neuron. Neuroscience, 489:176–184, May 2022. ISSN 03064522. doi: 10.1016/j.neuroscience.2021.07.008. URL https://linkinghub.elsevier.com/retrieve/pii/S0306452221003559. 34280492

[R79] Lampert Angelika and Korngreen Alon. Markov Modeling of Ion Channels. In Progress in Molecular Biology and Translational Science, volume 123, pages 1–21. Elsevier, 2014. ISBN 978–0-12–397897-4. doi: 10.1016/B978-0-12-397897-4.00009-7. URL https://linkinghub.elsevier.com/retrieve/pii/B9780123978974000097. 24560138

[R80] Sinha Ankur, Gleeson Padraig, Marin Bóris, Dura-Bernal Salvador, Panagiotou Sotirios, Crook Sharon, Cantarelli Matteo, Cannon Robert C., Davison Andrew P., Gurnani Harsha, and Silver R. Angus. The NeuroML ecosystem for standardized multi-scale modeling in neuroscience. eLife, May 2024. doi: 10.7554/eLife.95135.1. URL https://elifesciences.org/reviewed-preprints/95135v1. PMC11723582 39792574

[R81] Kumbhar Pramod, Hines Michael, Fouriaux Jeremy, Ovcharenko Aleksandr, King James, Delalondre Fabien, and Schürmann Felix. CoreNEURON : An Optimized Compute Engine for the NEURON Simulator. Frontiers in Neuroinformatics, 13:63, September 2019. ISSN 1662–5196. doi: 10.3389/fninf.2019.00063. URL https://www.frontiersin.org/article/10.3389/fninf.2019.00063/full. 31616273 PMC6763692

[R82] Zhang Yichen, He Gan, Ma Lei, Liu Xiaofei, Hjorth J. J. Johannes, Kozlov Alexander, He Yutao, Zhang Shenjian, Kotaleski Jeanette Hellgren, Tian Yonghong, Grillner Sten, Du Kai, and Huang Tiejun. A GPU-based computational framework that bridges neuron simulation and artificial intelligence. Nature Communications, 14(1):5798, September 2023. ISSN 2041–1723. doi: 10.1038/s41467-023-41553-7. URL https://www.nature.com/articles/s41467-023-41553-7. PMC10507119 37723170

[R83] Panagiotou Sotirios, Sidiropoulos Harry, Soudris Dimitrios, Negrello Mario, and Strydis Christos. EDEN: A High-Performance, General-Purpose, NeuroML-Based Neural Simulator. Frontiers in Neuroinformatics, 16:724336, May 2022. ISSN 1662–5196. doi: 10.3389/fninf.2022.724336. URL https://www.frontiersin.org/articles/10.3389/fninf.2022.724336/full. PMC9167055 35669596

[R84] Pagkalos Michalis, Chavlis Spyridon, and Poirazi Panayiota. Introducing the Dendrify framework for incorporating dendrites to spiking neural networks. Nature Communications, 14 (1):131, January 2023. ISSN 2041–1723. doi: 10.1038/s41467-022-35747-8. URL https://www.nature.com/articles/s41467-022-35747-8. PMC9832130 36627284

[R85] Hines Michael. NEURON and Python. Frontiers in Neuroinformatics, 3, 2009. ISSN 16625196. doi: 10.3389/neuro.11.001.2009. URL http://journal.frontiersin.org/article/10.3389/neuro.11.001.2009/abstract. PMC2636686 19198661

[R86] Mainen Zachary F. and Sejnowski Terrence J.. Influence of dendritic structure on firing pattern in model neocortical neurons. Nature, 382(6589):363–366, July 1996. ISSN 0028–0836, 1476–4687. doi: 10.1038/382363a0. URL https://www.nature.com/articles/382363a0. 8684467

[R87] Smith Spencer L., Smith Ikuko T., Branco Tiago, and Michael Häusser. Dendritic spikes enhance stimulus selectivity in cortical neurons in vivo. Nature, 503(7474):115–120, November 2013. ISSN 0028–0836, 1476–4687. doi: 10.1038/nature12600. URL https://www.nature.com/articles/nature12600. 24162850 PMC6319606

[R88] Petousakis Konstantinos-Evangelos, Park Jiyoung, Papoutsi Athanasia, Smirnakis Stelios, and Poirazi Panayiota. Modeling apical and basal tree contribution to orientation selectivity in a mouse primary visual cortex layer 2/3 pyramidal cell. eLife, 12:e91627, December 2023b. ISSN 2050–084X. doi: 10.7554/eLife.91627. URL https://elifesciences.org/articles/91627. 38054403 PMC10754496

[R89] Amitai Y., Friedman A., Connors B. W., and Gutnick M. J.. Regenerative Activity in Apical Dendrites of Pyramidal Cells in Neocortex. Cerebral Cortex, 3(1):26–38, 1993. ISSN 1047–3211, 1460–2199. doi: 10.1093/cercor/3.1.26. URL https://academic.oup.com/cercor/article-lookup/doi/10.1093/cercor/3.1.26. 8439739

[R90] González José. Distinguishing linear vs. non-linear integration in CA1 radial oblique dendrites: it’s about time. Frontiers in Computational Neuroscience, 5, 2011. ISSN 16625188. doi: 10.3389/fncom.2011.00044. URL http://journal.frontiersin.org/article/10.3389/fncom.2011.00044/abstract. PMC3214726 22171217

[R91] Virtanen Pauli, Gommers Ralf, Oliphant Travis E., and SciPy 1.0: Fundamental Algorithms for Scientific Computing in Python. Nature Methods, 17:261–272, 2020. doi: 10.1038/s41592-019-0686-2. 32015543 PMC7056644

